# Supplementary material for: Single-Molecule FRET Reveals Hidden Complexity in a Protein Energy Landscape
Source: Structure. 2015 Jan 6;23(1):190–8. doi: 10.1016/j.str.2014.10.023 (PMC4291146; doi:10.1016/j.str.2014.10.023)
Supplement: Document S1. Supplemental Experimental Procedures, Figures S1–S3, and Tables S1–S3 [file mmc1.pdf]

Structure, Volume *23*

Supplemental Information

Single-Molecule FRET Reveals Hidden Complexity in a Protein Energy Landscape

Maksym Tsytlonok, Shehu M. Ibrahim, Pamela J.E. Rowling, Wenshu Xu, Maria J. Ruedas-Rama, Angel Orte, David Klenerman, and Laura S. Itzhaki

Supplemental Information Tsytlonok *et al.*

Supplemental Experimental Procedures

Labeling

Before labeling the proteins, all buffers were sterile filtered and degassed. D34 was concentrated to 50-70 µM in buffer A (50 mM Tris-HCl pH 8, 50 mM NaCl) with 10 mM DTT. 2.5 ml of concentrated protein were loaded onto PD10 column and the protein was eluted with freshly degassed 3.5 ml of buffer A without DTT. The eluted D34 was first labeled with the acceptor Alexa Fluor 647 fluorophore (AF647) at a 1:1 ratio, followed by labeling with the donor Alexa Fluor 488 fluorophore (AF488) at the same ratio. The double-labeled D34 was separated from the homo-labeled and unlabeled species using ion-exchange chromatography (see Fig. S1A). Absorbance measurements on the double-labeled sample indicated that the labeling of the two dyes was approximately 50:50. The double-labeled D34 was analyzed by gel-filtration chromatography at 1 µM protein concentration and found to elute at a volume expected for the monomer without any evidence of oligomerization.

smFRET, Two-Color Coincidence Detection (TCCD) and Periodic Acceptor Excitation (PAX)

Briefly, excitation for TCCD measurements was achieved by focusing two overlapped laser beams (the 488-nm of a Spectra Physics Cyan CDRH laser at a power of 75 µW and a 633-nm line of a He-Ne laser at a power of 65 µW) inside the sample solution contained in a 8-well chambered cover glass, 7 µm above the glass surface using an oil immersion objective (Apochromat 60 x, NA 1.40, Nikon). smFRET measurements were performed using only the blue (488 nm) laser at a power of 75 µW on the same instrument. In both cases fluorescence emissions were separated into their donor and acceptor constituents and separately detected using two avalanche photodiodes. TCCD and smFRET data collected on each D34 sample were analysed using a common donor channel threshold value determined automatically by maximizing the association quotient in the TCCD experiment (Clarke et al., 2007), whereas the acceptor channel thresholds were independently determined. This ensured that both the smFRET and TCCD measurements of each sample resulted in approximately equal burst rates in the donor channel. All measurements were carried out at room temperature (23 ^{o}C).

Stability measurements of the cysteine mutants

In order to check that the cysteine mutations do not greatly perturb the stability of D34, ensemble urea-induced equilibrium denaturation experiments were performed monitoring intrinsic protein fluorescence (Fig. S1C). Fluorescence data were collected on a Perkin Elmer luminescence spectrometer LS55. Urea samples were prepared in the range of 0 M to 8 M using a Hamilton Microlab 500 Series, using buffer A (50 mM Tris-HCl pH 8, 50 mM NaCl) with 1 mM DTT. The samples were equilibrated at 25 °C for at least two hours before measurement. Previous measurements showed that the protein unfolds at equilibrium *via* a hyperfluorescent intermediate in which the C-terminal subdomain is structured and the N-terminal subdomain in unstructured (Werbeck et al., 2007). The *m*-values and midpoints of unfolding were determined by globally fitting the individual denaturation curves obtained from plotting the fluorescence intensity *versus* urea concentration at 0.5 nm intervals between 320 nm and 370 nm emission wavelength to the following three-state equation:

$$F = \frac{\alpha_N + \beta_N D + \exp\left(\frac{m_{N\to I}(D - D_{N\to I})}{RT}\right)\left(\alpha_U + \beta_U [D] \exp\left(\frac{m_{I\to U}(D - D_{I\to U})}{RT}\right)\right)}{1 + \exp\left(\frac{m_{N\to I}(D - D_{N\to I})}{RT}\right)\left(1 + \exp\left(\frac{m_{I\to U}(D - D^2_{I\to U})}{RT}\right)\right)},$$

where F is the observed fluorescence intensity; α_N is the fluorescence intensity of the native state at 0 M urea, β_N, the slope of the baseline for the native fluorescence, α_U is the fluorescence intensity of the denatured state with the sloping baseline β_U. D is the urea concentration and $D50_{N \to I}$ and $D50_{I \to U}$ are the midpoints for the unfolding transition between the native state and the intermediate and the intermediate and unfolded state, respectively, and $m_{I \to U}$ and $m_{I \to U}$ are the corresponding *m*-values. The *m* values and midpoints of unfolding were shared between the data sets; all other parameters were not constrained. Data were fitted to this equation using GraphPad Prism 5.0. The values obtained are listed in Table S1. Two of the three variants, ANK5-8 (which contains two mutations: C475S and S655C) and ANK9-12 (four mutations: C475S, C530S, S655C and S764C), showed small shifts (0.4 M for ANK5-8 and 0.2 M for ANK9-12) to lower urea concentrations in the midpoint of the first transition, which corresponds to the unfolding of the N-subdomain; the midpoint of the second transition, which corresponds to the unfolding of the C-subdomain, was the same as that of the wild type. ANK8-12 (four mutations: C475S, C530S, S655C and S780C, three of which are the same as those in ANK9-12) behaves like previously described destabilising mutations in the C-subdomain (1): the *m*-value for

the first transition is greater than that of the wild type, and for the second transition it is lower than that of wild type. In summary, the data show that the mutations have only small effects on the stability of D34, similar in magnitude to those previously observed for conservative valine-to-alanine mutations in this protein (Werbeck et al., 2007). For the truncated variant ANK9-12Δ, only a single transition is detected; it is difficult to interpret this transition in structural terms, as the signature hyperfluorescent intermediate is no longer present. The variant could not be purified in sufficient yield for further investigation of its stability (e.g. using equilibrium denaturation monitored by circular dichroism).

Stability measurements of labeled D34 wild type and variants

In order to check that the Alexa fluorophores (AF488 and AF647) did not disrupt the D34 structure, ensemble equilibrium denaturation experiments were performed on the double-labeled ANK3-5, ANK5-8, ANK8-12 and ANK9-12 proteins by exciting at 496 nm and collecting the emission spectra between 510 nm and 680 nm. Fluorescence intensity at 519 nm and 666 nm for donor and acceptor, respectively, were plotted as a function of urea concentration and the data were fitted to the following equation assuming a two-state behaviour:

$$F = \frac{(\alpha_{\mathrm{N}} + \beta_{\mathrm{N}} D) + (\alpha_{\mathrm{U}} + \beta_{\mathrm{U}} D)\exp\left(\frac{-m(\mathrm{D}_{50} - \mathrm{D})}{RT}\right)}{1 + \exp\left(\frac{-m(D_{50} - \mathrm{D})}{RT}\right)}$$

F is the observed fluorescence intensity; α_N is the native fluorescence signal at 0 M denaturant, β_N, the slope of the baseline for the native fluorescence, α_u is the denatured fluorescence with the sloping baseline for the denatured protein fluorescence β_u. D_{50} is the denaturant concentration at the midpoint for transition and m is the m-value.

For ANK3-5 and ANK9-12, we observed an increase in the fluorescence at 519 nm and a decrease in the fluorescence at 666 nm upon addition of urea, as expected for a decrease in FRET between the two fluorophores upon unfolding. The m-values and midpoints, obtained from fitting the data to the two-state equation, are listed in Table S2. The midpoints of unfolding are similar to those listed in Table S1 measured by intrinsic protein fluorescence for the unfolding of the relevant subdomain (N-subdomain in the case of ANK3-5 and C-subdomain in the case of

ANK9-12) in the unlabeled proteins, indicating that the dyes do not greatly perturb the stability of the protein. For ANK5-8 a single transition was observed at both wavelengths but the midpoints were slightly different for the two (Fig. S1D); the midpoint obtained at 519 nm is ~0.6 M higher than those of the two unfolding transitions of unlabelled ANK5-8, suggesting that this variant is slightly stabilised by the fluorophores. For ANK8-12 the fluorescence at 666 nm decreased upon unfolding, as observed for ANK3-5 and ANK9-12; however the fluorescence at 519 nm decreased slightly at ~2 M urea and then increased at higher urea concentrations. This behavior may reflect some local rearrangement of structure around the fluorophores before the full unfolding of the protein.

Time-resolved fluorescence measurements of labeled D34 variants, and relative orientation of the FRET-pair dyes

Fluorescence decay traces of single- and double-labeled D34 mutants in solution, at increasing concentrations of urea, were recorded in single photon timing (SPT) mode using a FluoTime 200 fluorescence lifetime spectrometer (PicoQuant GmbH, Berlin, Germany). The excitation source was either a 485-nm pulsed laser (LDH-P-C-485, PicoQuant) for the AF488 dye, or a 632-nm pulsed laser (LDH-P-C-635B, PicoQuant) for the AF647 dye. Both lasers were controlled by a PDL-800 driver unit (PicoQuant) using a 20 MHz repetition rate. The spectral bandwidth of the lasers was ± 5 nm and the temporal full width at half maxima of the pulses was lower than 90 ps. Fluorescence was collected after crossing through a polarizer set at the magic angle and a 2-nm bandwidth monocromator. The photon events are time stamped using a TimeHarp 200 PC-board (PicoQuant) and fluorescence decay histograms plotted over 1320 channels with a time increment per channel of 36 ps. Three fluorescence decay traces with 20,000 counts at the peak channel were collected for each dye at the following emission wavelengths: 515 nm, 525 nm, and 535 nm for the AF488, and 665, 670, and 675 nm for the AF647. Decay traces were analyzed by a least squares based deconvolution method in terms of multi-exponential functions, employing instrument response functions collected using Ludox scatterer. The three different traces were fitted globally with the decay times linked as shared parameters. The quality of the fits was judged by the value of the reduced χ^2 and the randomness in the distributions of weighted residuals and autocorrelation functions.

For the lifetime measurements we expressed and purified AF488 single-labeled variants with only one cysteine residue in each construct (referred to as C475,

C530, C655, C665, C764 and C780). Double-cysteine variants labeled with AF488 and AF647 were also analysed. The data are shown in figures S4 and S5.

Lifetime measurements of the AF488-labelled single-cysteine variants were performed as a function of urea concentration. This allows us to determine whether there are significant quenching effects from the neighbouring residues that could affect the FRET efficiency and thereby complicate the analysis. The lifetime for C764 (ANK 12) is unaltered upon unfolding (Fig. S2A). For C655 (ANK 8), C530 (ANK 5) and C780 (ANK12) the dye is slightly protected when folded and upon unfolding the lifetime decreases slightly, plateauing at ~3 M urea (Fig. S2A). The slight quenching of the dyes in these positions should not affect the FRET measurements. The largest effect of quenching is observed in C475 (a natural cysteine residue in D34, located in ANK 3) under native conditions. Although the quenching effect in this position is more noticeable than the others, the expected FRET towards the acceptor at cysteine C530 (the other natural cysteine residue) is much more efficient than the quenching. The competing nature of the two deactivation processes means that the faster process dominates, in this case the energy transfer. Therefore, the effect of quenching on the FRET efficiency is not very important.

The fluorescence lifetime of the AF647 dye in the folded and unfolded states was tested in the double-labelled FRET constructs (Fig. S2B). For ANK5-8 the lifetime is not altered upon unfolding. For ANK9-12 the acceptor dye, AF647, seems to be more protected in the folded state, and the lifetime is around 1.6 ns. Upon unfolding the lifetime decreases to 1.51 ns. In ANK3-5 and ANK8-12 the acceptor dye is slightly quenched in the folded state. The plots of the lifetimes together show that the environment of the dye is slightly different in the native states of different mutants, but once unfolded, AF647 has the same lifetime in all the mutants. In any case, the effects over the AF647 fluorescence lifetime are small, and the dye's ability to act as energy acceptor is not compromised by additional quenching effects.

The fluorescence lifetime of the AF488 energy donor in the double-labelled constructs was also measured as a function of urea concentration (Fig. S2C). In all constructs the donor dye AF488 is quenched by FRET when the protein is folded. The lifetime increases upon unfolding, as expected for the decreased FRET efficiency. The constructs ANK5-8 and ANK8-12 show lower quenching, consistent with the lower FRET efficiency and larger distance between the dyes. The lifetime of AF488 in the native states of ANK5-8 and ANK8-12 is 2.24 ns and 2.59 ns, respectively. Upon unfolding the lifetimes increase to 3.10 ns and 3.27 ns, respectively. These

values are lower than those of single-labeled mutants in 7 M urea (between 3.5 ns and 3.8 ns), suggesting that there is still some detectable FRET efficiency in the unfolded state. ANK9-12 and ANK3-5 show much more quenching, consistent with the high FRET in these pairs. The lifetime of AF488 in the native states of ANK9-12 and ANK3-5 is 1.01 ns and 0.97 ns, respectively. The lifetime increases upon unfolding to 3.15 ns and 2.70 ns, respectively. These values also show some detectable FRET efficiency in the unfolded state.

Due to the decrease of the donor fluorescence lifetime caused by energy transfer, the apparent FRET efficiency can be calculated from the average lifetimes. However, the double-labeled constructs have two different labeling positions for the donor and acceptor dyes, and a non-specific labeling strategy was used, so the donor fluorophore may be in different environments in different molecules. Assuming we have 50% of AF488 in one position and 50% in the other position, and given that we have measured the AF488 lifetimes in each position in the single-cysteine variants, then the average lifetime of AF488 in the absence of FRET can be determined from the average of the lifetimes obtained for the two AF488-labeled single-cysteine variants. This average lifetime in the absence of FRET (τ^{SL}) can then compared with the actual average lifetime of AF488 in the double-labeled construct (τ^{DL}) and the FRET efficiency calculated as: $E=1-(\tau^{DL})/(\tau^{SL})$. These results are shown in Figure S5.

Fluorescence anisotropy measurements were performed on a JASCO FP-6500 spectrofluorometer using a 450-W xenon lamp as the excitation source. Linearly polarized light of 488 and 633 nm was used for the excitation of the Alexa Fluor 488 and Alexa Fluor 647 respectively. The fluorescence anisotropy, r, is given by:

$$r = \frac{I_{VV} - G \times I_{VH}}{I_{VV} + 2G \times I_{VH}}$$

and the correction factor:

$$G = \frac{I_{HV}}{I_{HH}}$$

where the subscripts *V* and *H* represent respectively the vertical or horizontal position of the excitation (first subscript) or emission polarizer (second subscript). The obtained values of the fluorescence anisotropy are listed in Table S3.

The anisotropy values are employed to check whether the consideration of the κ^2 value of 2/3 (for random orientation of the dyes' dipoles in the FRET process) is correct into the estimation of the R_0 value. This is important to directly relate the FRET efficiency values with actual distances.

The Perrin equation relates the steady-state anisotropy of a fluorophore to the rotational correlation time and the lifetime of the dye. We have used this equation to estimate the limit anisotropy values for the fluorophores when attached rigidly to the protein. Considering a globular protein of 46.5 kDa, the rotational correlation time would be 18.1 ns. Applying the Perrin equation, the limit anisotropy (rigid dyes) would be 0.33, 0.35, and 0.37, respectively for fluorophores with a lifetime of 4 (AF488), 2.6 (AF488 in ANK3), and 1.5 ns (AF647). Hence, the anisotropy values of the AF488 constructs are in agreement with a depolarization occurring 5-10 fold faster than the rigid dye state. Similarly, the anisotropy values of the AF647 constructs are in agreement with a depolarization occurring 4-8 fold faster than the rigid dye state. However, the D34 is far from being a globular protein, and its shape is consistent with an even slower rotational diffusion. For a prolate ellipsoid-shaped hydrated protein with semi-axes of 65 Å and 20 Å, a much closer model for our D34 construct than the globular shape, the average of the three different rotational correlation times is 52.2 ns. With this rotational correlation time, the Perrin equation yields limit anisotropy values of 0.37, 0.38 and 0.39 for the three lifetimes mentioned above, respectively. In such case, the experimental anisotropy values indicate that the AF488 dye undergoes rotation 14-30 fold faster than the overall protein, and that the AF647 dye shows rotational diffusion 10-20 fold faster than the protein. These results are convincing enough to assume that the orientation of the dipoles in the FRET constructs can be considered random, and the κ^2 value of 2/3 is justified.

Table S1, related to Experimental Procedures. *m*-values and midpoints of unfolding (D50) for unlabeled wild-type D34 and variants, determined by globally fitting the denaturation curves obtained from plotting tryptophan fluorescence at 0.5 nm intervals between 320 nm and 370 nm emission wavelength to a three-state equation.

FRET variant	Mutations	$m_{N \to I}$ (kcal mol^{-1} M^{-1})	D50$_{N \to I}$ (M)	$m_{I \to U}$ (kcal mol^{-1} M^{-1})	D50$_{I \to U}$ (M)
ANK3-5	Wild type	3.46±0.75	2.64±0.06	1.69±0.26	2.32±0.11
ANK5-8	C475S, S655C	2.92±0.18	2.23±0.04	2.11±0.07	2.30±0.04
ANK8-12	C475S, C530S, S655C, S780C	5.80±0.35	2.40±0.02	0.71±0.08	2.00±0.04
ANK9-12	C475S, C530S, S665C, S764C	2.25±0.06	2.37±0.03	1.76±0.03	2.30±0.04
ANK9-12Δ*	C475S, C530S, S665C, S764C, S799stop	1.39±0.03	1.32±0.03		

*For ANK9-12Δ, only one transition could be clearly detected and therefore the data were fitted to a two-state equation.

Table S2, related to Figure 1. *m*-values and midpoints of unfolding (D50) for double-labeled wild-type D34 and variants, determined by fitting the denaturation curves obtained from plotting fluorescence intensity at emission wavelengths of 519 nm and 666 nm (for donor and acceptor dyes, respectively) upon excitation at 496 nm to a two-state equation.

Variant	*m* (519 nm) (kcalmol^{-1}M^{-1})	D50 (519 nm) (M)	*m* (666 nm) (kcalmol^{-1}M^{-1})	D50 (666 nm) (M)
ANK3-5	3.32±0.32	2.22±0.02	3.65±0.45	2.23±0.23
ANK5-8	2.88±0.78	2.88±0.07	2.27±0.26	2.28±0.04
ANK8-12*	-	-	2.10±0.18	2.30±0.04
ANK9-12	2.24±0.13	2.46±0.02	2.38±0.20	2.33±0.03
ANK9-12Δ*	4.04±2.0	1.95±0.23	-	-

*For ANK8-12, only the 666 nm data, for which there was a single transition, were fitted. For ANK9-12Δ, only the 519 nm data gave reliable fitting values, whereas the signal to noise for the 666 nm data was too low for a reliable fit.

Table S3, related to Figure 1. Anisotropy values of single- and double-labeled proteins.

Labeled residue	Location	*Anisotropy (r) donor*	*Anisotropy (r) acceptor*
C475, C530	ANK 3, 5	0.08	0.24
C530, C655	ANK 5, 8	0.17	0.25
C655, C780	ANK 8, 12	0.08	0.26
C665, C764	ANK 9, 12	0.13	0.30
C475	ANK 3	0.24	-
C530	ANK 5	0.16	-
C655	ANK 8	0.13	-
C665	ANK 9	0.13	-
C764	ANK 12	0.17	-
C780	ANK 12	0.12	-

A

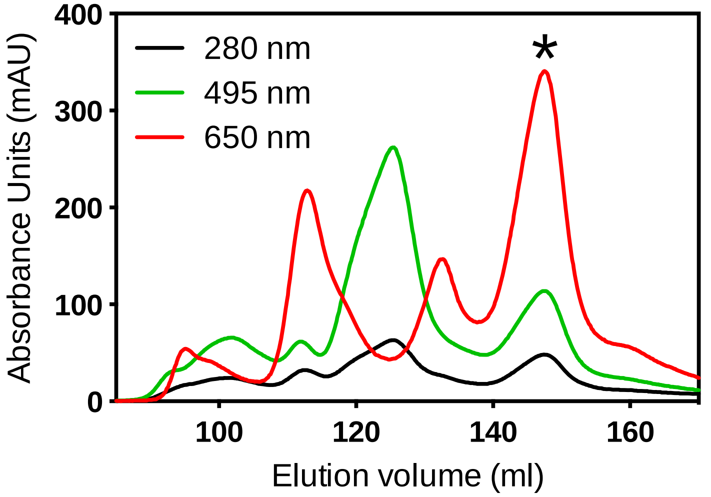

B

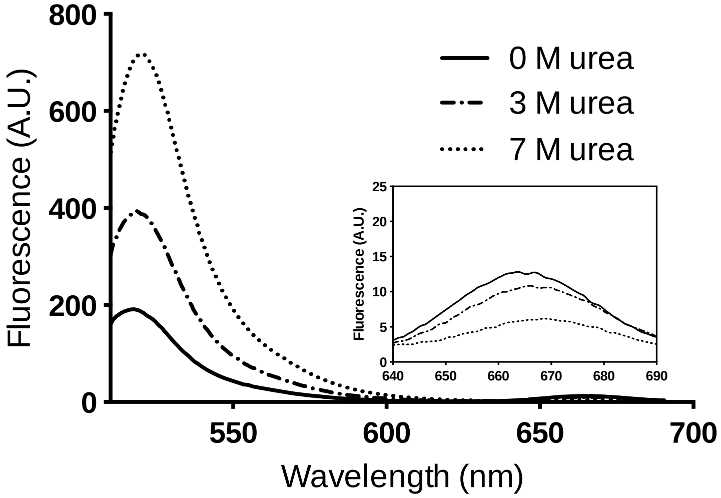

Figure S1, related to Experimental Procedures: Purification of AF488- and AF647-double-labelled wild-type D34.

(A) Wild-type D34 was labelled sequentially with AF647 and AF488. Unlabelled, homo-labelled (AF647 only or AF488 only) and hetero-labelled (AF488 and AF647 present) were separated by anion-exchange chromatography (MonoQ, GE Healthcare). The absorbance was followed at three wavelengths simultaneously (280 nm, 495 nm and 650 nm). The fractions containing D34 with both dyes were collected and pooled (indicated by a star).

(B) Fluorescence emission spectra of double-labeled wild-type D34 in 0 M, 3 M and 7 M urea using an excitation wavelength of 495 nm (inset shows the region around 666 nm magnified). Measurements were made at a protein concentration of 100 nM concentration in 50 mM Tris-HCl buffer pH 8, 150 mM NaCl and 1 mM TCEP at 25 °C.

C

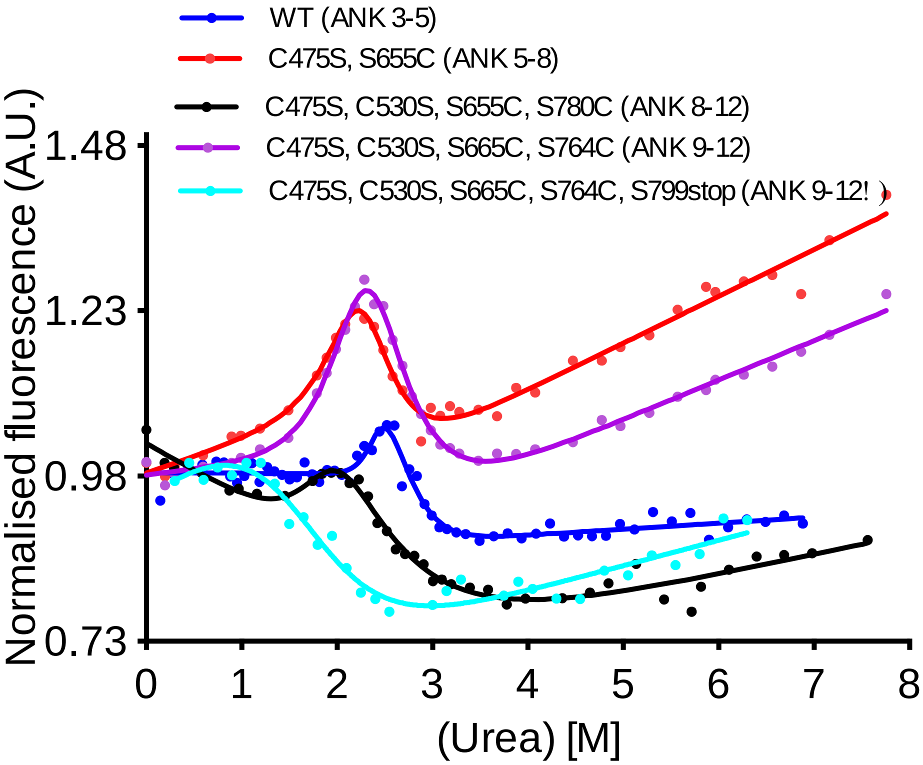

(C) Ensemble measurements of urea-induced denaturation for (unlabelled) D34 wild type and cysteine mutants monitored by tryptophan fluorescence. Denaturation profiles of WT (blue) and double-cysteine mutants of D34, monitored by fluorescence at 365 nm using an excitation wavelength of 280 nm. The data were collected at 1 µM protein concentration in 50 mM Tris pH-HCl buffer pH 8, 150 mM NaCl and 1 mM DTT at 25 °C. Wild-type D34 (WT) has cysteines at position 475 and 530 and is referred to here as ANK3-5 (blue); ANK5-8 has mutations C475S, S655C (red); ANK9-12 has C475S, C530S, S665C, S764C (purple); ANK8-12 has C475S, C530S, S655C, S780C (black). The denaturation curves were fitted to a three-state equation as described in the SI.

D

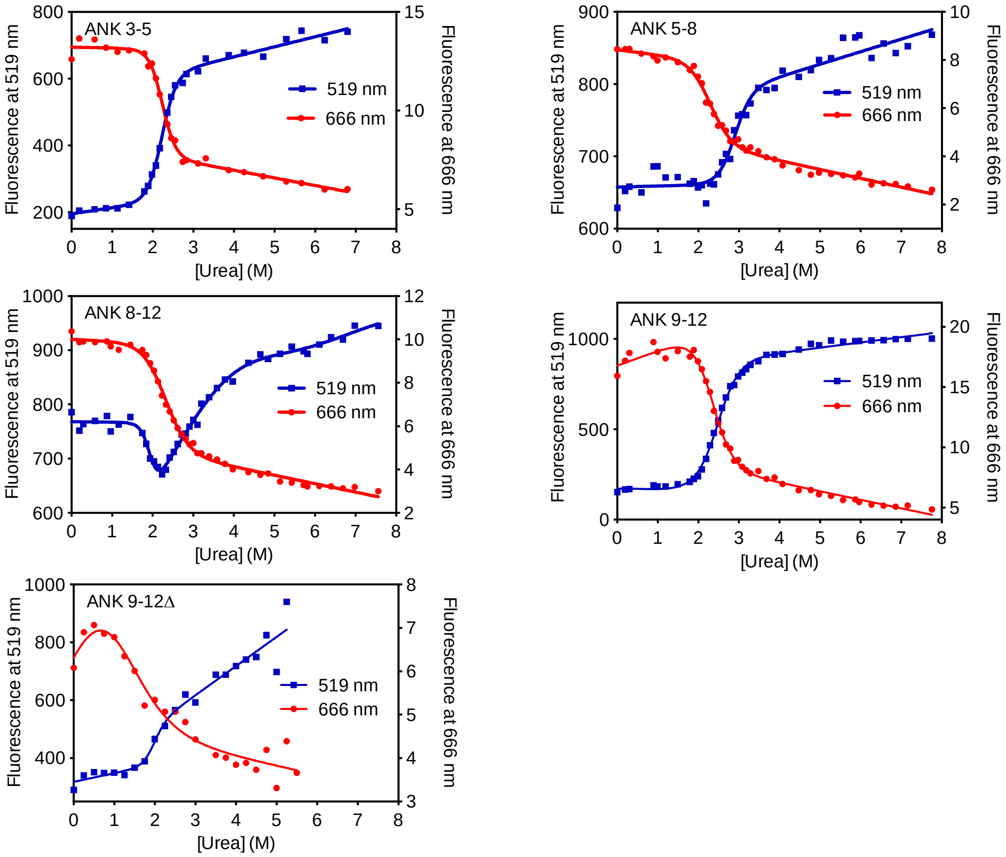

(D) Urea-induced denaturation profiles of D34 variants measured by AF488 and AF647 fluorescence. The data were collected at 0.2 μM protein concentration in 50 mM Tris-HCl buffer pH 8, 150 mM NaCl and 1 mM DTT at 25 °C. The denaturation curves were fitted to a two-state equation, with the exception of that of ANK8-12 at 519 nm, which was fitted to a three-state equation. The data for ANK9-12Δ at 666 nm gave high fitting errors due to the low signal-to-noise.

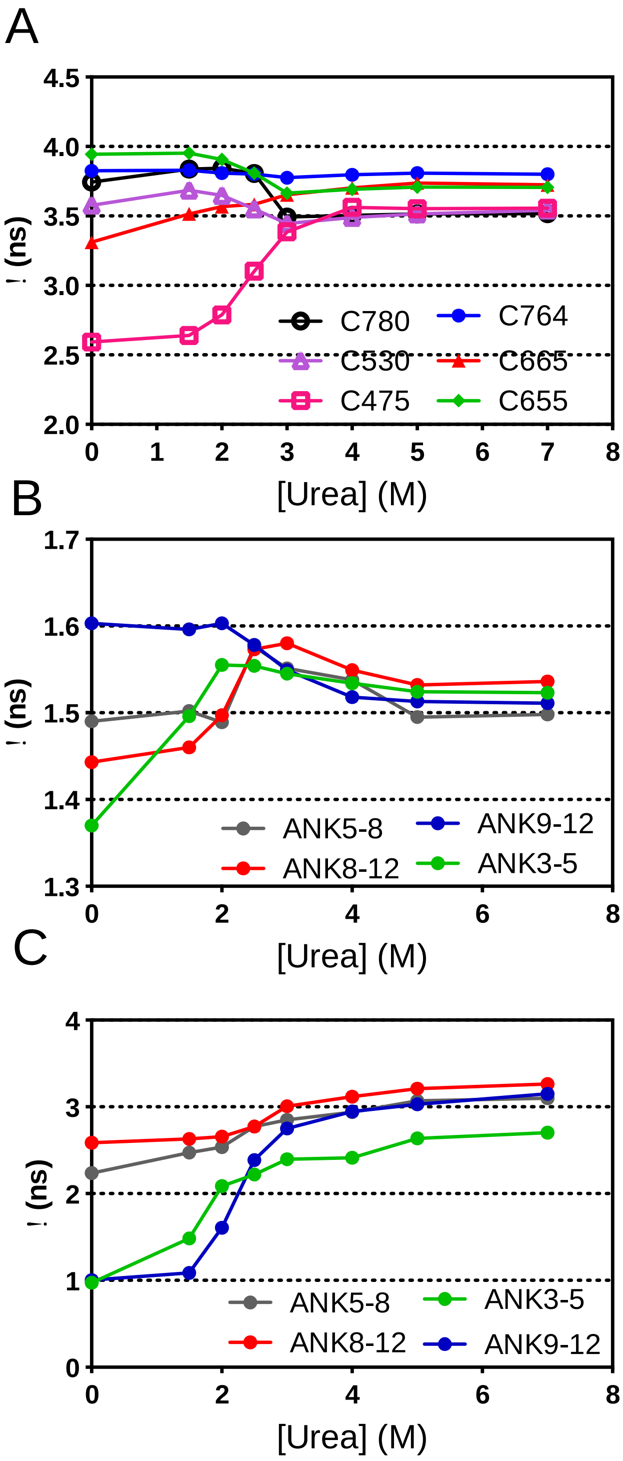

Figure S2, related to Figure 1. AF488 and AF647 fluorescence lifetimes in the single-labeled single-cysteine and double-labeled double-cysteine constructs as a function of increasing urea concentration

(A) AF488 fluorescence lifetimes in the single-cysteine constructs.

(B) AF647 and (C) AF488 fluorescence lifetimes in the double-cysteine constructs.

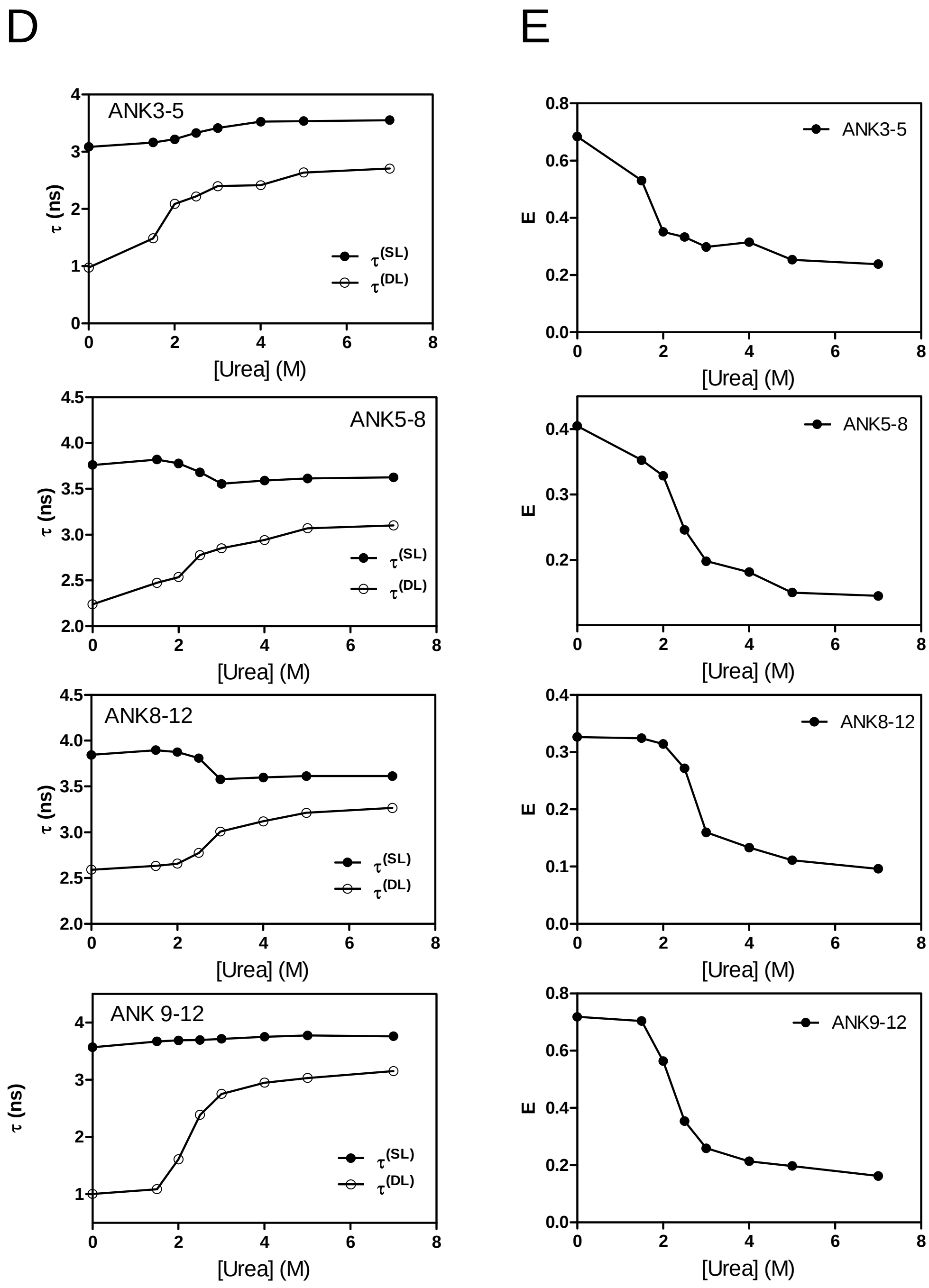

AF488 fluorescence lifetimes and FRET efficiencies of double-cysteine constructs as a function of urea concentration. (D) The average AF488 fluorescence lifetime τ^{SL} is the mean of the AF488 fluorescence lifetimes in the two single-labeled single-cysteine constructs. τ^{DL} is the AF488 fluorescence lifetime in the double-labeled double-cysteine constructs.

(E) The FRET efficiency, E, is calculated from τ^{SL} and τ^{DL} using the following equation: $E=1-(\tau^{DL})/(\tau^{SL})$.

A

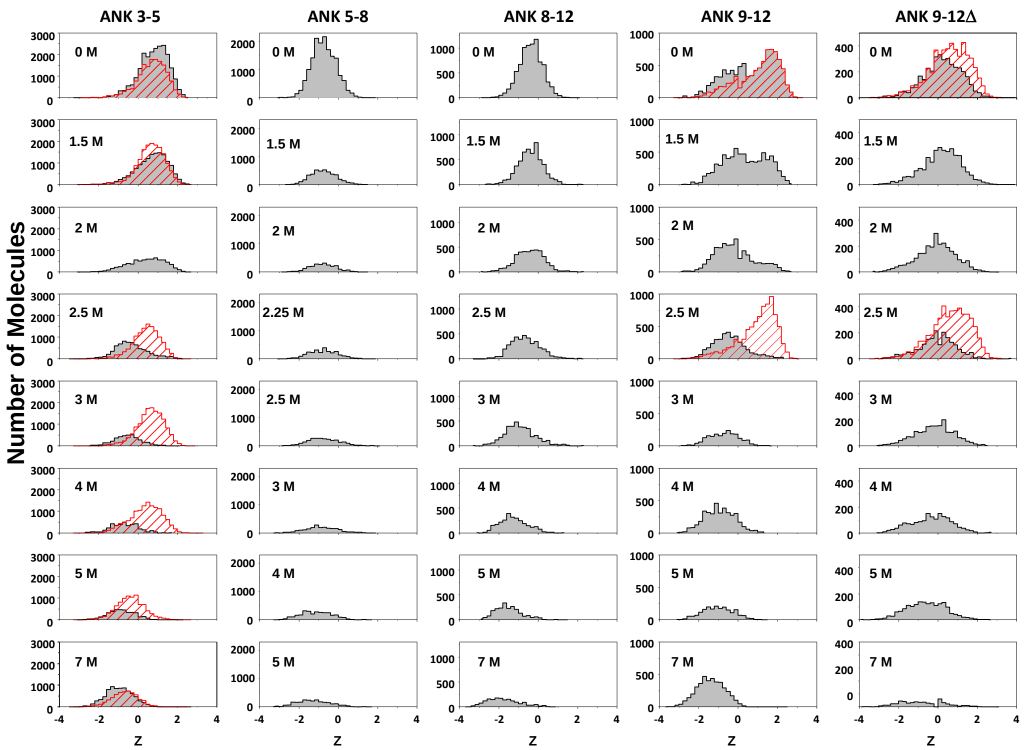

B

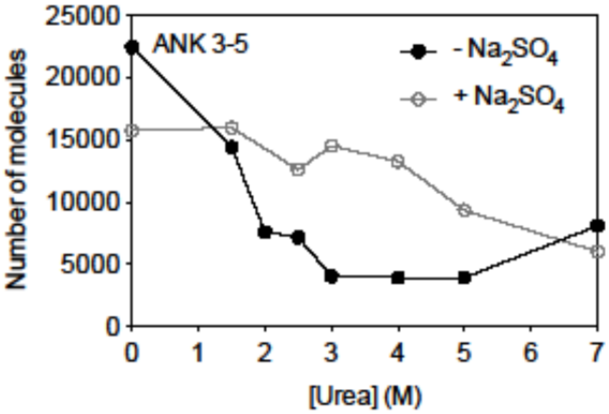

Figure S3, related to Figure 2. (A) Urea titrations of the FRET Z-parameter histograms of labelled wild-type D34 (ANK3-5) and variants. The data obtained for ANK3-5, ANK9-12 and ANK9-12Δ in the presence of 400 mM Na_2SO_4 are shown in red. (B) Comparison of the number of molecules as measured by 1 color FRET excitation *versus* urea concentration for ANK3-5 in the presence and absence of 400 mM Na_2SO_4.

References

Clarke, R.W., Orte, A., and Klenerman, D. (2007). Optimized threshold selection for single-molecule two-color fluorescence coincidence spectroscopy. Analytical chemistry *79*, 2771-2777.

Werbeck, N.D. and Itzhaki, L.S. (2007). Probing a moving target with a plastic unfolding intermediate of an ankyrin-repeat protein. Proceedings of the National Academy of Sciences of the United States of America *104*(19), 7863-7868.
