# Supplementary material for: Single-Molecule FRET Reveals Hidden Complexity in a Protein Energy Landscape
Source: Structure. 2015 Jan 6;23(1):190–8. doi: 10.1016/j.str.2014.10.023 (PMC4291146; doi:10.1016/j.str.2014.10.023)
Supplement: Document S2. Article plus Supplemental Information [file mmc2.pdf]

Structure
Short Article

# Single-Molecule FRET Reveals Hidden Complexity in a Protein Energy Landscape

Maksym Tsytlonok,[1,2,5,6] Shehu M. Ibrahim,[2,5] Pamela J.E. Rowling,[2,3] Wenshu Xu,[2,3] Maria J. Ruedas-Rama,[4] Angel Orte,[4] David Klenerman,[2,*] and Laura S. Itzhaki[2,3,*]
[1]MRC Cancer Cell Unit, Hutchison/MRC Research Centre, Hills Road, Cambridge CB2 0XZ, UK
[2]Department of Chemistry, University of Cambridge, Lensfield Road, Cambridge CB2 1EW, UK
[3]Department of Pharmacology, University of Cambridge, Tennis Court Road, Cambridge CB2 1PD, UK
[4]Department of Physical Chemistry, Faculty of Pharmacy, Campus Cartuja, University of Granada, 18071 Granada, Spain
[5]Co-first author
[6]Present address: Vlaams Instituut voor Biotechnologie, Vrije Universiteit Brussel, Pleinlaan 2, 1050 Brussels, Belgium
*Correspondence: dk10012@cam.ac.uk (D.K.), lsi10@cam.ac.uk (L.S.I.)
http://dx.doi.org/10.1016/j.str.2014.10.023
This is an open access article under the CC BY license (http://creativecommons.org/licenses/by/3.0/).

## SUMMARY

**Here, using single-molecule FRET, we reveal previously hidden conformations of the ankyrin-repeat domain of AnkyrinR, a giant adaptor molecule that anchors integral membrane proteins to the spectrin-actin cytoskeleton through simultaneous binding of multiple partner proteins. We show that the ankyrin repeats switch between high-FRET and low-FRET states, controlled by an unstructured "safety pin" or "staple" from the adjacent domain of AnkyrinR. Opening of the safety pin leads to unravelling of the ankyrin repeat stack, a process that will dramatically affect the relative orientations of AnkyrinR binding partners and, hence, the anchoring of the spectrin-actin cytoskeleton to the membrane. Ankyrin repeats are one of the most ubiquitous molecular recognition platforms in nature, and it is therefore important to understand how their structures are adapted for function. Our results point to a striking mechanism by which the order-disorder transition and, thereby, the activity of repeat proteins can be regulated.**

## INTRODUCTION

Single-molecule measurements can resolve the complexity that is inherent in protein energy landscapes but obscured in ensemble experiments because of sample averaging. Such studies of small single-domain proteins and miniproteins have yielded fundamental insights into folding mechanisms (Chung and Eaton, 2013; Ferreon et al., 2009; Kuzmenkina et al., 2005; Laurence et al., 2005; Nath et al., 2012; Nettels et al., 2007; Sherman and Haran, 2006). In other respects, however, new information will be limited if single-molecule analysis is applied only to small proteins because such proteins generally populate just two states, the native and denatured states, and, therefore, all that can be observed is the exchange between them. Large, multidomain proteins have been subjected to little single-molecule folding experiments other than force-probe characterization of their mechanics (Bertz et al., 2010; Kotamarthi et al., 2013; Shank et al., 2010; Wang et al., 2012). Tandem-repeat proteins such as ankyrin, tetratricopeptide, and HEAT repeats will be particularly interesting targets for single-molecule analysis because of a fundamental property distinguishing them from globular proteins, namely the copopulation of multiple, partly folded intermediates under equilibrium unfolding conditions and the accessibility of multiple pathways in the unfolding kinetics (Ferreiro et al., 2005; Lowe and Itzhaki, 2007; Tripp and Barrick, 2008; Werbeck and Itzhaki, 2007; Werbeck et al., 2008). This feature arises from the modular nature of repeat-protein structures and the high level of similarity between the modules at both sequence and structural levels. However, it is very difficult to detect such degeneracy experimentally because of limitations of scale; small repeat proteins have been the focus of most studies to date, and yet giant repeat proteins have much greater potential for heterogeneity. We reasoned, therefore, that single-molecule Förster resonance energy transfer (smFRET) between a series of fluorescent donor-acceptor dye pairs along the repeat stack would allow direct visualization of the spectrum of conformations accessible to this special class of proteins.

D34 is a 426-residue 12-ankyrin-repeat fragment from the giant multidomain protein AnkyrinR (Michaely et al., 2002) (Figure 1A), one of a family of molecular adaptors that mediate the attachment of integral membrane proteins to the spectrin-actin cytoskeleton (Bennett and Healy, 2009). The membrane-binding domain of AnkyrinR forms a superhelical spiral comprising 24 ankyrin repeats that can be divided into four subdomains, D1–D4 (of which D34 is subdomains D3 and D4). Experimental and computational studies suggest that ion transporters bind on one side of the 24-ankyrin repeat stack and that clathrin and cell adhesion molecules bind on the other side (Grey et al., 2012; Kim et al., 2011; Michaely and Bennett, 1995; Michaely et al., 2002). The ankyrin-repeat membrane-binding domain is followed by the spectrin-binding domain (SBD), and an extended loop of the latter domain is found to pack against the five most C-terminal ankyrin repeats in the crystal structure of D34 (Figure 1A). It has been proposed that the interaction between the two domains serves to orientate the multiple AnkyrinR binding partners relative to one another and, thereby, position the spectrin-actin cytoskeleton to the membrane surface.

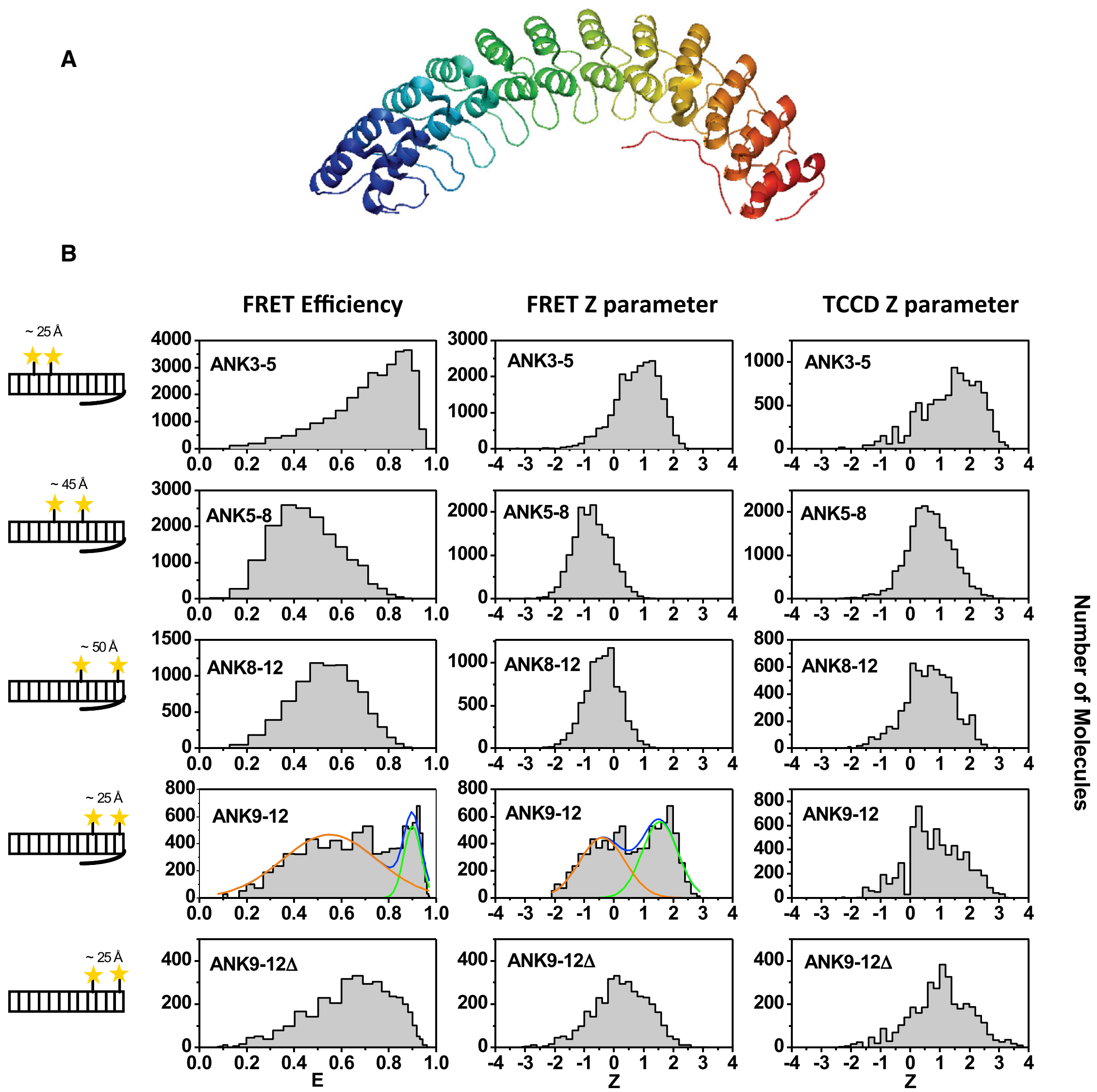


**Figure 1. FRET Efficiency and Z Parameter Histograms of the Labeled Wild-Type D34 and Variants under Native Conditions**

(A) Schematic of the D34 structure.

(B) Locations of the Alexa dye pairs in wild-type D34 (ANK3-5) and the other variants are shown schematically to the left of the histograms. FRET efficiency histograms (left histograms) were obtained by single (blue) laser excitation of the double-labeled samples. FRET efficiency, E, was calculated as $E = I_A / (\gamma I_D + I_A)$, where $I_D$ and $I_A$ are the donor (blue channel) and acceptor (red channel) fluorescence intensities, respectively, and $\gamma$ is the instrument factor determined previously as 0.54. FRET Z parameter histograms (middle histograms) of the same data set are presented in the form $Z = \ln(I_A / I_D)$. TCCD Z parameter histograms (right histograms) were obtained by simultaneous two-laser (blue and red) excitation of the double-labeled samples. The data were acquired at 25 pM protein concentration in 50 mM Tris-HCl buffer (pH 8), 150 mM NaCl, and 0.001% Tween 20 at 25°C. The orange and green lines on the FRET efficiency histogram and FRET Z parameter histogram of ANK9-12 are the Gaussian fits of the data at low FRET and high FRET, respectively.

Previous ensemble experiments showed that D34 unfolds via an intermediate state in which the six C-terminal ankyrin repeats are folded and the six N-terminal ankyrin repeats are unfolded (Werbeck and Itzhaki, 2007; Werbeck et al., 2008). The data also hinted tantalizingly at the presence of other partly folded states under mildly denaturing conditions. Here, smFRET analysis reveals a previously hidden complexity in the conformational ensemble of D34. Under native conditions, we find that, although the N-terminal subdomain of D34 is compact and homogeneously folded, the C-terminal subdomain populates two states in roughly equal proportions, one having high FRET and the other low FRET. Upon truncation of the SBD loop, the high-FRET state is no longer detected, and only the low-FRET state is observed, indicating that the loop acts as a molecular "staple" to control the structure of the C-terminal repeats. The results show that both folded and partially unfolded forms of the protein are present even under native conditions, adding another layer of complexity to the already rich spectrum of conformational states

that make up the energy landscape of D34 and pointing to an interesting mechanism by which the folding and, thereby, the function of tandem-repeat proteins can be regulated. This mechanism could be also be exploited to regulate the binding properties of designed ankyrin-repeat proteins, which are currently in development as alternatives to therapeutic antibodies (Stumpp and Amstutz, 2007).

## RESULTS

### Design of D34 Variants along the Repeat Array

D34 comprises residues 402–827 from AnkyrinR, corresponding to the 12 C-terminal ankyrin repeats of the 24-ankyrin-repeat membrane-binding domain and an unstructured loop from the SBD that folds back onto the ankyrin repeats (Figure 1A). It has two solvent-exposed cysteine residues at positions 475 and 530 (the residues numbers used here correspond to those in the full-length protein) in the third and fifth ankyrin repeats, respectively (ANK3-5), whose Cα atoms are separated by 25 Å and which we used for labeling with the FRET dye pair. To monitor the behavior of the C-terminal repeats and of the repeats in the middle of the protein, three further constructs were designed in which one or both of the wild-type cysteine residues were mutated to serine, and serine residues at other positions were mutated to cysteine: ANK5-8 (having cysteines at positions 530 and 655), ANK8-12 (C655 and C780), and ANK9-12 (C665 and C764), with 45, 50, and 25 Å between the Cα atoms of the two cysteine residues, respectively (Figure 1). The stabilities of the double-cysteine variants were determined using urea-induced unfolding measured by tryptophan fluorescence, and they were found to be only slightly less stable than the wild-type (see Supplemental Experimental Procedures, Figure S1C, and Table S1, available online, for further details). Next, the effect of labeling on the stability of the wild-type and the double-cysteine variants was analyzed using urea-induced unfolding and measuring of donor and acceptor fluorescence, and the labeling was found to have only small effects on stability (see Supplemental Experimental Procedures, Figure S1D, and Table S2 for further details).

We tested the integrity of the fluorescence emission of the dyes in the FRET construct by measuring the fluorescence lifetime of the fluorophores (Supplemental Experimental Procedures and Figure S2). The Förster's distance value for the FRET pair was then calculated to be 56 Å (Invitrogen), if random orientation of the dyes' dipoles ($\kappa^2$ = 2/3) is assumed. We then confirmed this assumption by measuring the fluorescence anisotropies for each double-cysteine variant and all labeled single-cysteine variants (Table S3). The values are notably lower than the limiting anisotropy, indicating that the orientation of the FRET-pair dyes can be considered random.

### Single-Molecule FRET and TCCD Measurements Reveal Heterogeneity in the C-Terminal Subdomain of D34, with Both Compact and Noncompact States Copopulated under Native Conditions

We applied two-color coincidence detection (TCCD)/FRET to probe the conformations of ANK3-5, ANK5-8, ANK8-12, and ANK9-12. In TCCD experiments, two (blue and red) overlapping laser beams simultaneously excited a dilute (50 pM) solution of the D34 molecules as they diffused through the confocal volume one molecule at a time. Donor and acceptor fluorescence bursts are detected in coincidence when they originate from the same molecule, therefore allowing direct quantification of the number of molecules with both donor and acceptor fluorophores on them. In FRET, the donor fluorophores were excited with only the blue laser to measure the separation between the dye pairs because the measured simultaneous bursts depend on the conformation of an individual species as it passes through the confocal volume.

Data analysis for both TCCD and FRET measurements was carried out using a coincidence criterion described in detail previously (Orte et al., 2008; Ye et al., 2012). The data for each D34 sample were analyzed using a common donor channel threshold value determined automatically by maximizing the association quotient in the TCCD experiment (Clarke et al., 2007), whereas the acceptor channel thresholds were determined independently. This ensured that both the FRET and TCCD measurements of each sample resulted in approximately equal burst rates in the donor channel. The combined use of TCCD and FRET measurements allows the estimation of the proportion of molecules that were in non-FRET conformations. The use of the coincidence technique allows the detection of both compact (high-FRET) and noncompact (low-FRET) species while isolating singly labeled D34 molecules from the data. Different conformations are detectable, and their relative changes in the population as conditions are changed can be evaluated.

smFRET experiments were performed first under native conditions (50 mM Tris-HCl buffer (pH 8), 150 mM NaCl, 1 mM EDTA, and 0.001% Tween20). The FRET efficiency histogram for wild-type D34 (ANK3-5) is shown in Figure 1B (ANK3-5 FRET Efficiency). The high FRET efficiency is consistent with the small distance between the cysteine residues in the crystal structure (25 Å between the two Cα atoms). FRET efficiency histograms are not well described by Gaussian distributions, especially at very high or very low FRET efficiencies (Wallace et al., 2000). FRET data are better represented by a lognomal distribution using the Z parameter defined as $Z = \ln(I_A/I_D)$, where $I_A$ and $I_D$ are the acceptor and donor fluorescence intensities, respectively, having been corrected to account for background and crosstalk. The Z parameter histograms for FRET and TCCD measurements of ANK3-5 are shown in Figure 1B. The TCCD Z parameter histogram shows a relatively broad distribution of events. In contrast, the FRET Z parameter histogram measured by single-laser (blue) excitation shows one narrow peak with Z values around 1 (corresponding to a FRET efficiency of 0.83), suggesting that there is a homogeneous population for ANK3-5 under native conditions. It should be noted that the lifetime measurements of single-cysteine variants labeled with the donor only show partial quenching of the dye at position C475 when the protein is under native conditions (Supplemental Experimental Procedures and Figure S2). This additional quenching could potentially contribute to heterogeneity and broadening of the single-molecule FRET histograms. However, given that the FRET efficiency under these conditions is high, the energy transfer is the faster of the two processes (energy transfer and quenching), and, therefore, the quenching does not compete.

The FRET efficiency histogram of ANK5-8 shows a broad distribution of events peaking at a low efficiency of ~0.4 (Figure 1B). This result was not surprising given that the two cysteine residues are separated by a relatively large distance (45 Å between the Cα atoms). As with ANK3-5, the TCCD and FRET data for ANK5-8 are better represented by Z parameter histograms. The FRET and TCCD Z parameter histograms each show one peak at Z values of approximately −1 (a FRET efficiency of 0.4) and 0.5, respectively. The distance between the two cysteine residues in ANK8-12 (50 Å) is slightly larger than that in ANK5-8 (45 Å). The FRET efficiency histogram for ANK8-12 shows a unimodal distribution of events with a peak at FRET efficiency of 0.55 (Figure 1B), which is somewhat higher than that observed for ANK5-8. The higher FRET efficiency despite a larger interdye distance may simply reflect the likely dynamic nature of the protein structure compared with the one that is "frozen" in the crystal structure. Specifically, previous folding studies by our group (Werbeck and Itzhaki, 2007; Werbeck et al., 2008) as well as limited proteolysis experiments (Michaely and Bennett, 1993) and steered molecular dynamics simulations (Sotomayor et al., 2005) have shown that each six-repeat half of D34 is a stable subdomain and that the interaction between the two subdomains (i.e., between ANK6 and ANK7) is disrupted first before global unfolding occurs. The dye pair in the ANK5-8 construct span these two subdomains, and if, as suggested by these previous studies, there is some unzipping of the intersubdomain interface occurring even under native conditions, then we would expect, on average, the dyes to be further apart than what the static distance measured in the crystal structure indicates. In contrast, the dyes in ANK8-12 lie within a single subdomain, and, therefore, their separation may be more accurately reflected by the crystallographic values.

The two cysteine residues of ANK9-12 are in close proximity (25 Å), like those of ANK3-5. However, the histograms obtained for ANK9-12 are very different from those for ANK3-5 (Figure 1B). The FRET efficiency histogram for ANK9-12 can be fitted to a high-FRET subpopulation at a value of 0.9 and a low-FRET subpopulation at 0.55. Moreover, in contrast to the FRET Z histograms obtained for ANK3-5 and ANK5-8, the ANK9-12 FRET Z parameter histogram shows two clear subpopulations at −0.1 (a FRET efficiency of 0.6) and 1.5 (a FRET efficiency of 0.9), in agreement with the FRET efficiency histogram. In summary, the FRET efficiency and Z parameter histograms suggest that the N-terminal subdomain of D34 is homogenous and compactly folded, whereas the C-terminal subdomain is heterogeneous under native conditions, with a subpopulation of approximately 50% that is not compactly folded. Unlike ANK9-12, no high-FRET population is observed or expected for ANK8-12 because the separation of the dyes is much greater. We focused most of our subsequent analysis on the two variants, ANK3-5 and ANK9-12, in which the cysteine residues are sufficiently close for there to be high-FRET species.

## The C-Terminal Subdomain Becomes More Compact upon Addition of Sodium Sulfate

Kosmotrope $Na_2SO_4$ has been found to stabilize the compact states of proteins (e.g., Pugh et al., 2010). We therefore examined the effect of $Na_2SO_4$ on the conformational ensemble of D34 using the ANK3-5 and ANK9-12 proteins. In red, in Figure S3A, we show their FRET Z parameter histograms in the presence of 400 mM $Na_2SO_4$. $Na_2SO_4$ does not have a significant effect on the histogram of ANK3-5. For ANK9-12, by contrast, there is a significant shift in molecules from the low-FRET subpopulation to the high-FRET subpopulation that is most evident in the TCCD histogram. Now the ratio of low-FRET to high-FRET species is ~30:70 rather than ~50:50. These experiments are consistent with the population of a noncompact state for the C-terminal subdomain of D34 under native conditions (discussed further subsequently).

## Single-Molecule Equilibrium Denaturation Measurements by TCCD Detects All Events in the Denatured State

Single-molecule urea-induced equilibrium unfolding experiments were performed next (see Figure 2 for low and high urea concentrations and Figure S3 for the complete set of urea concentrations). As discussed earlier, the histograms do not follow simple Gaussian distribution, and, therefore, the Z parameter histogram was applied. For ANK3-5, the FRET Z parameter histogram, which shows a peak at a Z value of ~1 at 0 M urea, becomes broader at urea concentrations of 1.5, 2, and 2.5 M. These data can be fitted to Gaussian with two peaks at Z values around 1 and −1. Above 3 M urea, only the peak with the Z value of −1 is observed. These results are consistent with unfolding of the protein leading to separation of the dye pairs. The transition between high and low Z values is similar to the unfolding transition measured by dye fluorescence in bulk experiments (Figure S1D; Table S2) and is also in agreement with the lifetime measurements as a function of urea (Figure S2). The results are consistent with a cooperative mode of unfolding for the N-terminal subdomain of D34, as observed previously in ensemble experiments (Werbeck and Itzhaki, 2007).

We noticed that the total number of FRET events decreased with increasing urea concentration (see Figure 2B, 1c). To establish the origins of this behavior, we next performed equilibrium denaturation experiments with TCCD using two-laser (blue and red) excitation of the samples. The advantage of TCCD is that, by exciting both dyes simultaneously and applying the coincidence criteria, molecules with very low FRET efficiencies are not thresholded out of the analysis. Therefore, TCCD permits the assessment of the number of dual-labeled molecules, even those showing very low FRET efficiency (less than 0.2). Using TCCD, we now observe that the total number of molecules is constant and does not change with urea concentration (Figure 2B, 2c). Therefore, the decrease in the number of FRET events compared with the number of TCCD events clearly indicates that the unfolding is increasing the population of a very low FRET state. This was confirmed by measurement of the FRET efficiency of the unfolded protein using time-resolved fluorescence lifetime measurements (Figure S2E), which showed FRET efficiencies below 0.25 at 7 M urea for all of the constructs. The same behavior was also observed for ANK5-8, ANK8-12, and ANK9-12 (Figure 2B). Urea denaturation experiments were also performed in the presence of 400 mM $Na_2SO_4$ (red histograms in Figure S3A). With $Na_2SO_4$, the urea-dependent

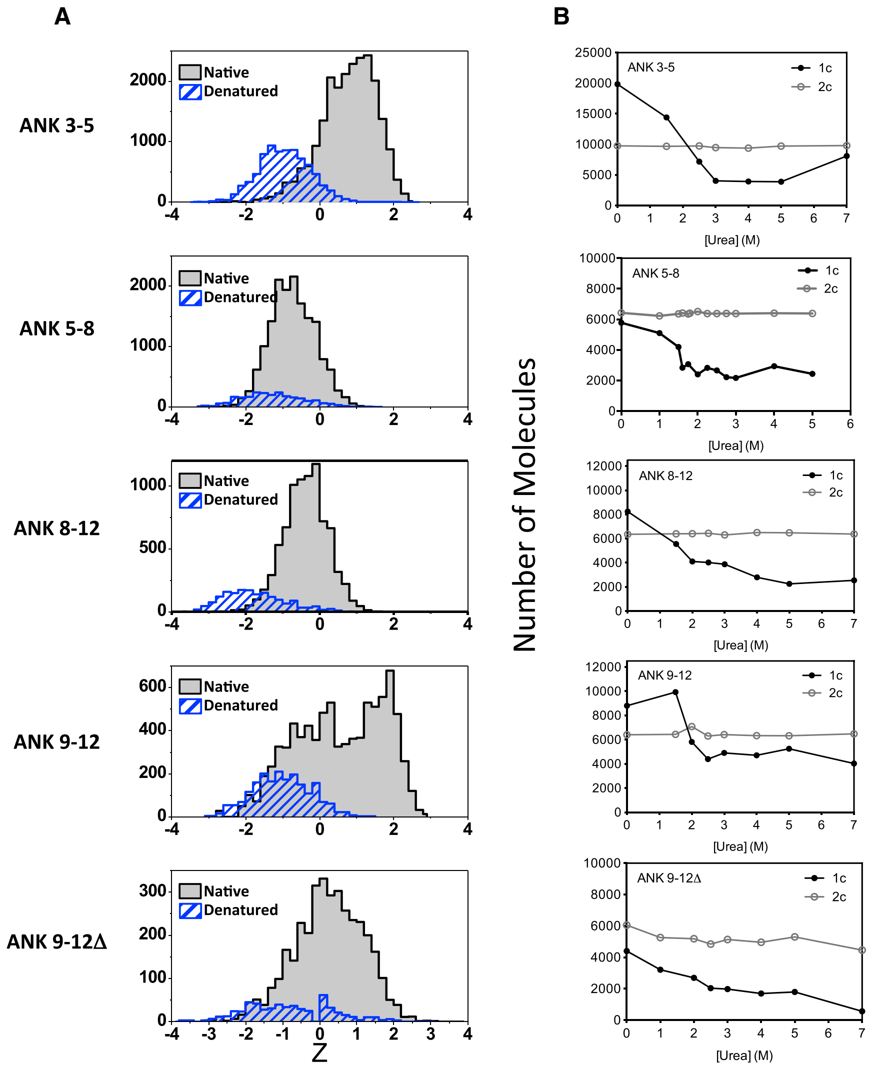


**Figure 2. Effects of Urea on the FRET Z Parameter Histograms of the Labeled Wild-Type D34 and Variants**

(A) FRET Z parameter histograms are shown in the absence of urea (gray) and in the presence of 7 M urea (blue). The full urea titrations are shown in Figure S6.

(B) The number of molecules as measured by smFRET using single (blue) laser excitation (1c) and TCCD using dual laser (blue and red) excitation (2c) are plotted as a function of urea concentration.

decrease in the number of FRET events is shifted to a higher urea concentration (Figure S3B), consistent with the stabilizing effect of $Na_2SO_4$ on D34.

### Deletion of the C-Terminal Tail Results in Only the Low-FRET State for the C-Terminal Subdomain

We speculated that the conformational heterogeneity observed for the C-terminal subdomain under native conditions is related to the unstructured SBD loop (tail) that is packed against the five most C-terminal ankyrin repeats of D34 in the crystal structure (Figure 1A). To investigate the role of the tail, we made a truncated variant of ANK9-12 in which the tail was deleted at residue S799, ANK9-12Δ. The truncated variant is destabilized relative to the nontruncated variant (Figure S1C), as shown previously (Werbeck and Itzhaki, 2007). We confirmed, by circular dichroism and analytical gel filtration, that the protein is monomeric and retains a substantial helical structure and compactness, and, therefore, we can assume that at least the N-terminal subdomain is not perturbed by the deletion of the C-terminal tail. The protein was labeled successfully, and the FRET Z parameter histogram is shown in Figure 1B. It is strikingly different from that of ANK9-12 because only a low-FRET state is observed for ANK9-12Δ, and there is no high-FRET state detectable. Moreover, upon addition of $Na_2SO_4$, the histogram is shifted to higher FRET, as also observed for ANK9-12 (Figure S3). These results are consistent with the hypothesis that the tail exists in two different conformations, one packed against the ankyrin repeats, resulting in a fully folded and compact C-terminal subdomain, and the other unpacked from the ankyrin repeats, resulting in partial unfolding of the ankyrin repeats and to an expanded form. The kosmotrope $Na_2SO_4$ shifts the equilibrium between these two conformations to the more compact one.

## DISCUSSION

We used single-molecule FRET to systematically probe the energy landscape of D34. To quantify the changes in the populations of the different FRET states at increasing denaturant concentrations, we used both TCCD with two-laser excitation of the samples and smFRET using only single-laser (blue) excitation. Using the TCCD method, we found that the total number of molecules was constant across the range of urea concentrations, whereas this number decreased gradually with increasing urea concentration when smFRET measurement was used. These results underline the importance of using the two techniques in combination to fully understand the biophysical properties of a protein because TCCD permits the assessment of the number of dual-labeled molecules, even those showing very low FRET.

Previous ensemble analysis showed that the unfolding of D34 is a complex and multistep process in which the unfolding of the N-terminal subdomain occurs cooperatively, whereas the C-terminal subdomain unravels in a noncooperative manner (Werbeck and Itzhaki, 2007). Moreover, protein engineering suggest that mutations modulate the energy landscape of D34 so that different unfolding intermediates become dominant. The single-molecule analysis described here adds a

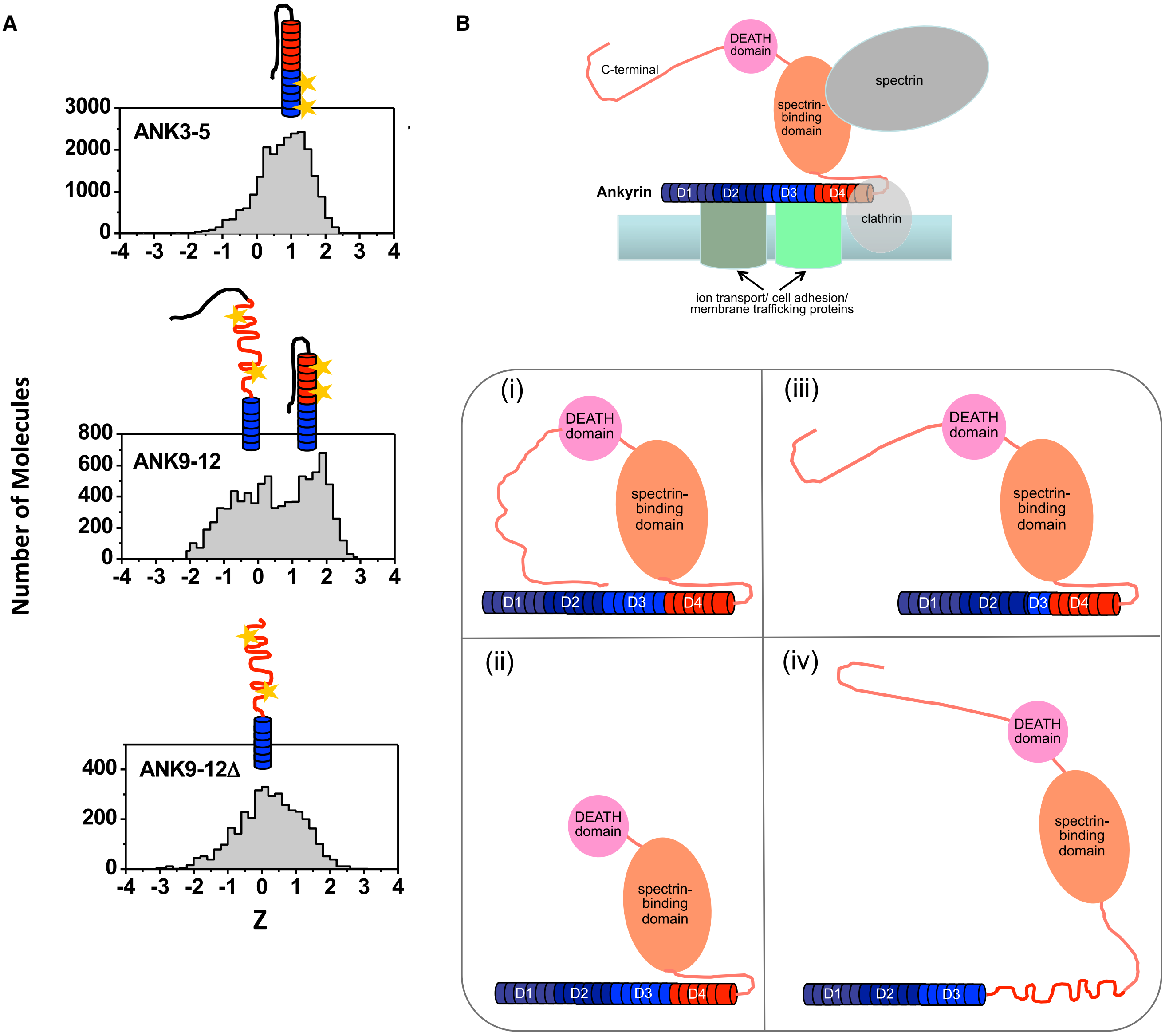


**Figure 3. Schematics Summarizing the Main Findings of the Single-Molecule Analysis and Their Functional Implications**

(A) Schematic of the energy landscape of D34 resolved by smFRET. The unstructured loop can adopt two different arrangements under native conditions, giving rise to distinct FRET populations.

(B) Schematic showing the mechanisms by which Ankyrins are auto-regulated. (Top) Ankyrin domain structure and binding of partner proteins. The MBD comprises 24 ankyrin repeats in four subdomains, D1–D4, the C-terminal 12 of which constitute D34. For simplicity, the ankyrin repeats are drawn with a linear shape, although they in fact form a superhelical spiral. The MBD and SBD are intimately associated via an unstructured loop from the latter domain that packs back against the six most C-terminal ankyrin repeats of the former domain. (Bottom) Multiple mechanism operate to regulate and diversify Ankyrin functions: (i) intramolecular association of the C-terminal regulatory domain and the MBD; alternative splicing, which, for example, leads to deletion of (ii) the C-terminal regulatory domain or (iii) individual ankyrin repeats; and (iv) folding/unfolding of the C-terminal ankyrin repeats, controlled by the unstructured segment from the SBD acting as a molecular safety pin or staple, which will further modulate the anchoring activity of Ankyrins.

further layer of complexity. The results show that the "native state" of D34 is not a homogeneous species. Although the N-terminal subdomain is homogeneous and compactly folded, there is a heterogeneous ensemble of conformations of the C-terminal subdomain. Two roughly equal populations are present under native conditions, one with a high FRET efficiency and one with a low FRET efficiency. Moreover, a variant in which the protein is truncated before the tail showed only the low-FRET species. These results indicate that the low-FRET population arises from the detachment of the unstructured tail from the C-terminal ankyrin repeats and consequent unravelling of the ankyrin stack (Figure 3A).

The properties of D34 revealed here are particularly significant in view of the biological function of Ankyrins (Figure 3B).

The three vertebrate Ankyrins, AnkyrinR, AnkyrinB, and AnkyrinG, are critical for normal physiology, and dysfunction is associated with numerous human diseases, including cardiac and neurological disorders (Bennett and Healy, 2009). They are very large proteins comprising an N-terminal membrane-binding domain (MBD), a central SBD, a death domain of poorly defined function, and a highly variable C-terminal regulatory domain. The MBD is composed of 24 ankyrin repeats forming a superhelical spiral that can be divided into four subdomains, D1–D4. D34 corresponds to D3 and D4 from AnkyrinR. The MBD and SBD are not independent of one another. Rather, they interact via an unstructured segment from the latter domain that was found in the crystal structure of D34 to fold back and pack against the D4 subdomain (Michaely et al., 2002), and a similar "linker" has been identified in AnkyrinB and AnkyrinG and has been shown to modulate the membrane-protein recognition of the ankyrin repeats. By binding membrane-spanning proteins via the ankyrin repeats of the MBD and spectrin via the SBD, Ankyrins anchor the membrane surface to the spectrin-actin cytoskeleton. There are no structures of the ankyrin repeats in complex with their binding partners. However, computational and experimental analyses suggest that the exceptionally long groove of the ankyrin superhelix allows a diverse range of membrane proteins, including ion transport, cell adhesion, and membrane trafficking molecules, to bind (Davis et al., 1989; Grey et al., 2012; Kim et al., 2011; Michaely and Bennett, 1995; Michaely et al., 2002). Ankyrins appear to have evolved an extraordinarily complex set of autoregulatory mechanisms that may work in a combinatorial fashion to coordinate the activities of the different domains, modulating subcellular localization and membrane protein-binding specificities and, thereby, greatly diversifying their functions (Figure 3B). First, the C-terminal regulatory domain interacts intramolecularly with the MBD to negatively modulate the latter's affinity for its binding partners through an as yet unknown allosteric mechanism (Davis et al., 1992; Figure 3B, i). Second, the *Ank* genes are subject to varied and tissue-specific mRNA splicing events that result in heterogeneous populations of Ankyrin polypeptides (Cunha et al., 2008; Hall and Bennett, 1987; Lux et al., 1990). Notable are splicing that removes the C-terminal regulatory domain, leading to alleviation of its repressive effect on the MBD (Figure 3B, ii), and splicing within the MBD that removes exons encoding individual ankyrin repeats, which will alter the molecular recognition capabilities of this domain (Figure 3B, iii).

Our single-molecule analysis reveals a third, distinct mechanism by which Ankyrin function can be regulated (Figure 3B, iv). We show that the unstructured segment of the SBD acts as a staple or safety pin that induces the folding of the ankyrin repeats of the D4 subdomain. Under native conditions, this safety pin is in the open position for ~50% of the molecules (and likely at an even higher percentage at body temperature), and the ankyrin repeats are consequently partly unfolded, a process that will dramatically affect the positioning of membrane proteins to the spectrin-based cytoskeleton. The disordered nature of these ankyrin repeats will also modulate the interactions of the MBD with its binding partners. Interestingly, alternative exons have been identified close to this region between the MBD and SBD, which would further modulate the properties of the ankyrin repeats (Cunha et al., 2008; Hall and Bennett, 1987; Lux et al., 1990).

The absence of sequence-distant contacts likely affords repeat proteins extreme flexibility and particular molecular recognition capabilities (Ferreiro et al., 2005; Sivanandan and Naganathan, 2013), consistent with the proposal that they are a distinct class midway between globular structured proteins and intrinsically disordered proteins (Forwood et al., 2010). An example is IκBα, the two C-terminal ankyrin repeats of which possess features of intrinsic disorder (Lamboy et al., 2011, 2013). These repeats are found to fold upon binding to its partner, NFκB (Truhlar et al., 2006). A distinct way to regulate the order-disorder transition of repeat proteins is revealed in our study of AnkyrinR. Here it is the intramolecular association of the C-terminal ankyrin repeats with the adjacent domain that induces folding, thereby providing an additional level of control with which this complex adaptor molecule anchors membrane proteins to the underlying cytoskeleton.

## EXPERIMENTAL PROCEDURES

### Production of Dye-Labeled D34 Wild-Type and Mutants

Site-directed mutagenesis of D34 and expression and purification of wild-type and mutants were performed as described previously (Werbeck and Itzhaki, 2007). Wild-type D34 has two solvent-exposed cysteine residues at positions C475 and C530 (the residues numbers used here correspond to those in the full-length protein) in the third and fifth ankyrin repeats (referred to as ANK3-5). Labeling with Alexa Fluor 488 and Alexa Fluor 647 was performed as described in the Supplemental Experimental Procedures. The bulk fluorescence behavior of the double-labeled protein upon excitation at 485 nm was analyzed as a function of urea concentration (Supplemental Experimental Procedures). The fluorescence intensity of the donor increased and that of the acceptor decreased with increasing urea concentration (Figures S1B and S1D), as expected for a decrease in FRET between the two fluorophores upon unfolding. Next, mutant variants were created with one or both of the wild-type cysteine residues mutated to serine and cysteine residues introduced at other positions to probe other regions of the structure. These were expressed, purified, and labeled as for the wild-type. They showed similar bulk fluorescence behavior as the wild-type (Figure S1D). Anisotropy and fluorescence lifetime measurements were performed to check that the labeled proteins were suitable for single-molecule FRET measurements (Supplemental Experimental Procedures).

### Single-Molecule Fluorescence Measurements: smFRET, TCCD, and PAX

smFRET, dual laser excitation in TCCD, and periodic acceptor excitation (PAX) measurements were performed using a homebuilt laser confocal microscope. The instrumentation, data acquisition, and data analysis have been described in detail previously, including validation of the TCCD/FRET measurements by PAX (Ye et al., 2012), and are summarized in the Supplemental Experimental Procedures. PAX measurements were performed on ANK9-12 to confirm that there was no threshold bias in the TCCD/FRET measurements and similar FRET efficiencies and populations were obtained.

## SUPPLEMENTAL INFORMATION

Supplemental Information includes Supplemental Experimental Procedures, three figures, and three tables and can be found with this article online at http://dx.doi.org/10.1016/j.str.2014.10.023.

## AUTHOR CONTRIBUTIONS

L.S.I. and D.K. conceived the research. M.T., S.M.I., P.J.E.R., W.X., and M.J.R. performed research and analyzed data. M.T., S.M.I., A.O., D.K., and L.S.I. wrote the paper.

## ACKNOWLEDGMENTS

L.S.I., M.T., S.M.I., P.J.E.R., and W.X. acknowledge support from the Medical Research Council of the UK (including Grant G1002329), and L.S.I. acknowledges support from the Medical Research Foundation.

Received: May 18, 2014
Revised: October 1, 2014
Accepted: October 6, 2014
Published: December 31, 2014

## REFERENCES

Bennett, V., and Healy, J. (2009). Membrane domains based on ankyrin and spectrin associated with cell-cell interactions. Cold Spring Harb. Perspect. Biol. *1*, a003012.

Bertz, M., Chen, J., Feige, M.J., Franzmann, T.M., Buchner, J., and Rief, M. (2010). Structural and mechanical hierarchies in the alpha-crystallin domain dimer of the hyperthermophilic small heat shock protein Hsp16.5. J. Mol. Biol. *400*, 1046–1056.

Chung, H.S., and Eaton, W.A. (2013). Single-molecule fluorescence probes dynamics of barrier crossing. Nature *502*, 685–688.

Clarke, R.W., Orte, A., and Klenerman, D. (2007). Optimized threshold selection for single-molecule two-color fluorescence coincidence spectroscopy. Anal. Chem. *79*, 2771–2777.

Cunha, S.R., Le Scouarnec, S., Schott, J.J., and Mohler, P.J. (2008). Exon organization and novel alternative splicing of the human ANK2 gene: implications for cardiac function and human cardiac disease. J. Mol. Cell. Cardiol. *45*, 724–734.

Davis, L., Lux, S.E., and Bennett, V. (1989). Mapping the ankyrin-binding site of the human erythrocyte anion exchanger. J. Biol. Chem. *264*, 9665–9672.

Davis, L.H., Davis, J.Q., and Bennett, V. (1992). Ankyrin regulation: an alternatively spliced segment of the regulatory domain functions as an intramolecular modulator. J. Biol. Chem. *267*, 18966–18972.

Ferreiro, D.U., Cho, S.S., Komives, E.A., and Wolynes, P.G. (2005). The energy landscape of modular repeat proteins: topology determines folding mechanism in the ankyrin family. J. Mol. Biol. *354*, 679–692.

Ferreon, A.C., Gambin, Y., Lemke, E.A., and Deniz, A.A. (2009). Interplay of alpha-synuclein binding and conformational switching probed by single-molecule fluorescence. Proc. Natl. Acad. Sci. USA *106*, 5645–5650.

Forwood, J.K., Lange, A., Zachariae, U., Marfori, M., Preast, C., Grubmüller, H., Stewart, M., Corbett, A.H., and Kobe, B. (2010). Quantitative structural analysis of importin-$\beta$ flexibility: paradigm for solenoid protein structures. Structure *18*, 1171–1183.

Grey, J.L., Kodippili, G.C., Simon, K., and Low, P.S. (2012). Identification of contact sites between ankyrin and band 3 in the human erythrocyte membrane. Biochemistry *51*, 6838–6846.

Hall, T.G., and Bennett, V. (1987). Regulatory domains of erythrocyte ankyrin. J. Biol. Chem. *262*, 10537–10545.

Kim, S., Brandon, S., Zhou, Z., Cobb, C.E., Edwards, S.J., Moth, C.W., Parry, C.S., Smith, J.A., Lybrand, T.P., Hustedt, E.J., and Beth, A.H. (2011). Determination of structural models of the complex between the cytoplasmic domain of erythrocyte band 3 and ankyrin-R repeats 13-24. J. Biol. Chem. *286*, 20746–20757.

Kotamarthi, H.C., Sharma, R., Narayan, S., Ray, S., and Ainavarapu, S.R. (2013). Multiple unfolding pathways of leucine binding protein (LBP) probed by single-molecule force spectroscopy (SMFS). J. Am. Chem. Soc. *135*, 14768–14774.

Kuzmenkina, E.V., Heyes, C.D., and Nienhaus, G.U. (2005). Single-molecule Forster resonance energy transfer study of protein dynamics under denaturing conditions. Proc. Natl. Acad. Sci. USA *102*, 15471–15476.

Lamboy, J.A., Kim, H., Lee, K.S., Ha, T., and Komives, E.A. (2011). Visualization of the nanospring dynamics of the IkappaBalpha ankyrin repeat domain in real time. Proc. Natl. Acad. Sci. USA *108*, 10178–10183.

Lamboy, J.A., Kim, H., Dembinski, H., Ha, T., and Komives, E.A. (2013). Single-molecule FRET reveals the native-state dynamics of the I$\kappa$B$\alpha$ ankyrin repeat domain. J. Mol. Biol. *425*, 2578–2590.

Laurence, T.A., Kong, X., Jäger, M., and Weiss, S. (2005). Probing structural heterogeneities and fluctuations of nucleic acids and denatured proteins. Proc. Natl. Acad. Sci. USA *102*, 17348–17353.

Lowe, A.R., and Itzhaki, L.S. (2007). Rational redesign of the folding pathway of a modular protein. Proc. Natl. Acad. Sci. USA *104*, 2679–2684.

Lux, S.E., John, K.M., and Bennett, V. (1990). Analysis of cDNA for human erythrocyte ankyrin indicates a repeated structure with homology to tissue-differentiation and cell-cycle control proteins. Nature *344*, 36–42.

Michaely, P., and Bennett, V. (1993). The membrane-binding domain of ankyrin contains four independently folded subdomains, each comprised of six ankyrin repeats. J. Biol. Chem. *268*, 22703–22709.

Michaely, P., and Bennett, V. (1995). The ANK repeats of erythrocyte ankyrin form two distinct but cooperative binding sites for the erythrocyte anion exchanger. J. Biol. Chem. *270*, 22050–22057.

Michaely, P., Tomchick, D.R., Machius, M., and Anderson, R.G. (2002). Crystal structure of a 12 ANK repeat stack from human ankyrinR. EMBO J. *21*, 6387–6396.

Nath, A., Sammalkorpi, M., DeWitt, D.C., Trexler, A.J., Elbaum-Garfinkle, S., O'Hern, C.S., and Rhoades, E. (2012). The conformational ensembles of $\alpha$-synuclein and tau: combining single-molecule FRET and simulations. Biophys. J. *103*, 1940–1949.

Nettels, D., Gopich, I.V., Hoffmann, A., and Schuler, B. (2007). Ultrafast dynamics of protein collapse from single-molecule photon statistics. Proc. Natl. Acad. Sci. USA *104*, 2655–2660.

Orte, A., Clarke, R.W., and Klenerman, D. (2008). Fluorescence coincidence spectroscopy for single-molecule fluorescence resonance energy-transfer measurements. Anal. Chem. *80*, 8389–8397.

Pugh, S.D., Gell, C., Smith, D.A., Radford, S.E., and Brockwell, D.J. (2010). Single-molecule studies of the Im7 folding landscape. J. Mol. Biol. *398*, 132–145.

Shank, E.A., Cecconi, C., Dill, J.W., Marqusee, S., and Bustamante, C. (2010). The folding cooperativity of a protein is controlled by its chain topology. Nature *465*, 637–640.

Sherman, E., and Haran, G. (2006). Coil-globule transition in the denatured state of a small protein. Proc. Natl. Acad. Sci. USA *103*, 11539–11543.

Sivanandan, S., and Naganathan, A.N. (2013). A disorder-induced domino-like destabilization mechanism governs the folding and functional dynamics of the repeat protein I$\kappa$B$\alpha$. PLoS Comput. Biol. *9*, e1003403.

Sotomayor, M., Corey, D.P., and Schulten, K. (2005). In search of the hair-cell gating spring elastic properties of ankyrin and cadherin repeats. Structure *13*, 669–682.

Stumpp, M.T., and Amstutz, P. (2007). DARPins: a true alternative to antibodies. Curr. Opin. Drug Discov. Devel. *10*, 153–159.

Tripp, K.W., and Barrick, D. (2008). Rerouting the folding pathway of the Notch ankyrin domain by reshaping the energy landscape. J. Am. Chem. Soc. *130*, 5681–5688.

Truhlar, S.M., Torpey, J.W., and Komives, E.A. (2006). Regions of IkappaBalpha that are critical for its inhibition of NF-kappaB.DNA interaction fold upon binding to NF-kappaB. Proc. Natl. Acad. Sci. USA *103*, 18951–18956.

Wallace, M.I., Ying, L., Balasubramanian, S., and Klenerman, D. (2000). FRET Fluctuation Spectroscopy: Exploring the Conformational Dynamics of a DNA Hairpin Loop. J. Phys. Chem. B *104*, 11551–11555.

Wang, Y., Chu, X., Suo, Z., Wang, E., and Wang, J. (2012). Multidomain protein solves the folding problem by multifunnel combined landscape: theoretical investigation of a Y-family DNA polymerase. J. Am. Chem. Soc. *134*, 13755–13764.

Werbeck, N.D., and Itzhaki, L.S. (2007). Probing a moving target with a plastic unfolding intermediate of an ankyrin-repeat protein. Proc. Natl. Acad. Sci. USA *104*, 7863–7868.

Werbeck, N.D., Rowling, P.J., Chellamuthu, V.R., and Itzhaki, L.S. (2008). Shifting transition states in the unfolding of a large ankyrin repeat protein. Proc. Natl. Acad. Sci. USA *105*, 9982–9987.

Ye, Y., Blaser, G., Horrocks, M.H., Ruedas-Rama, M.J., Ibrahim, S., Zhukov, A.A., Orte, A., Klenerman, D., Jackson, S.E., and Komander, D. (2012). Ubiquitin chain conformation regulates recognition and activity of interacting proteins. Nature *492*, 266–270.

**Structure, Volume *23***

## Supplemental Information

# Single-Molecule FRET Reveals Hidden Complexity in a Protein Energy Landscape

**Maksym Tsytlonok, Shehu M. Ibrahim, Pamela J.E. Rowling, Wenshu Xu, Maria J. Ruedas-Rama, Angel Orte, David Klenerman, and Laura S. Itzhaki**

# Supplemental Information Tsytlonok *et al.*

## Supplemental Experimental Procedures

*Labeling*

Before labeling the proteins, all buffers were sterile filtered and degassed. D34 was concentrated to 50-70 μM in buffer A (50 mM Tris-HCl pH 8, 50 mM NaCl) with 10 mM DTT. 2.5 ml of concentrated protein were loaded onto PD10 column and the protein was eluted with freshly degassed 3.5 ml of buffer A without DTT. The eluted D34 was first labeled with the acceptor Alexa Fluor 647 fluorophore (AF647) at a 1:1 ratio, followed by labeling with the donor Alexa Fluor 488 fluorophore (AF488) at the same ratio. The double-labeled D34 was separated from the homo-labeled and unlabeled species using ion-exchange chromatography (see Fig. S1A). Absorbance measurements on the double-labeled sample indicated that the labeling of the two dyes was approximately 50:50. The double-labeled D34 was analyzed by gel-filtration chromatography at 1 μM protein concentration and found to elute at a volume expected for the monomer without any evidence of oligomerization.

*smFRET, Two-Color Coincidence Detection (TCCD) and Periodic Acceptor Excitation (PAX)*

Briefly, excitation for TCCD measurements was achieved by focusing two overlapped laser beams (the 488-nm of a Spectra Physics Cyan CDRH laser at a power of 75 μW and a 633-nm line of a He-Ne laser at a power of 65 μW) inside the sample solution contained in a 8-well chambered cover glass, 7 μm above the glass surface using an oil immersion objective (Apochromat 60 x, NA 1.40, Nikon). smFRET measurements were performed using only the blue (488 nm) laser at a power of 75 μW on the same instrument. In both cases fluorescence emissions were separated into their donor and acceptor constituents and separately detected using two avalanche photodiodes. TCCD and smFRET data collected on each D34 sample were analysed using a common donor channel threshold value determined automatically by maximizing the association quotient in the TCCD experiment (Clarke et al., 2007), whereas the acceptor channel thresholds were independently determined. This ensured that both the smFRET and TCCD measurements of each sample resulted in approximately equal burst rates in the donor channel. All measurements were carried out at room temperature (23 $^{o}$C).

*Stability measurements of the cysteine mutants*

In order to check that the cysteine mutations do not greatly perturb the stability of D34, ensemble urea-induced equilibrium denaturation experiments were performed monitoring intrinsic protein fluorescence (Fig. S1C). Fluorescence data were collected on a Perkin Elmer luminescence spectrometer LS55. Urea samples were prepared in the range of 0 M to 8 M using a Hamilton Microlab 500 Series, using buffer A (50 mM Tris-HCl pH 8, 50 mM NaCl) with 1 mM DTT. The samples were equilibrated at 25 °C for at least two hours before measurement. Previous measurements showed that the protein unfolds at equilibrium *via* a hyperfluorescent intermediate in which the C-terminal subdomain is structured and the N-terminal subdomain in unstructured (Werbeck et al., 2007). The *m*-values and midpoints of unfolding were determined by globally fitting the individual denaturation curves obtained from plotting the fluorescence intensity *versus* urea concentration at 0.5 nm intervals between 320 nm and 370 nm emission wavelength to the following three-state equation:

$$F = \frac{\alpha_N + \beta_N D + \exp\left(\frac{m_{N\to I}(D - D_{N\to I})}{RT}\right)\left(\alpha_U + \beta_U [D] \exp\left(\frac{m_{I\to U}(D - D_{I\to U})}{RT}\right)\right)}{1 + \exp\left(\frac{m_{N\to I}(D - D_{N\to I})}{RT}\right)\left(1 + \exp\left(\frac{m_{I\to U}(D - D_{I\to U}^2)}{RT}\right)\right)},$$

where $F$ is the observed fluorescence intensity; $\alpha_N$ is the fluorescence intensity of the native state at 0 M urea, $\beta_N$, the slope of the baseline for the native fluorescence, $\alpha_U$ is the fluorescence intensity of the denatured state with the sloping baseline $\beta_U$. D is the urea concentration and $D50_{N \to I}$ and $D50_{I \to U}$ are the midpoints for the unfolding transition between the native state and the intermediate and the intermediate and unfolded state, respectively, and $m_{I \to U}$ and $m_{I \to U}$ are the corresponding *m*-values. The *m* values and midpoints of unfolding were shared between the data sets; all other parameters were not constrained. Data were fitted to this equation using GraphPad Prism 5.0. The values obtained are listed in Table S1. Two of the three variants, ANK5-8 (which contains two mutations: C475S and S655C) and ANK9-12 (four mutations: C475S, C530S, S655C and S764C), showed small shifts (0.4 M for ANK5-8 and 0.2 M for ANK9-12) to lower urea concentrations in the midpoint of the first transition, which corresponds to the unfolding of the N-subdomain; the midpoint of the second transition, which corresponds to the unfolding of the C-subdomain, was the same as that of the wild type. ANK8-12 (four mutations: C475S, C530S, S655C and S780C, three of which are the same as those in ANK9-12) behaves like previously described destabilising mutations in the C-subdomain (1): the *m*-value for

the first transition is greater than that of the wild type, and for the second transition it is lower than that of wild type. In summary, the data show that the mutations have only small effects on the stability of D34, similar in magnitude to those previously observed for conservative valine-to-alanine mutations in this protein (Werbeck et al., 2007). For the truncated variant ANK9-12Δ, only a single transition is detected; it is difficult to interpret this transition in structural terms, as the signature hyperfluorescent intermediate is no longer present. The variant could not be purified in sufficient yield for further investigation of its stability (e.g. using equilibrium denaturation monitored by circular dichroism).

*Stability measurements of labeled D34 wild type and variants*

In order to check that the Alexa fluorophores (AF488 and AF647) did not disrupt the D34 structure, ensemble equilibrium denaturation experiments were performed on the double-labeled ANK3-5, ANK5-8, ANK8-12 and ANK9-12 proteins by exciting at 496 nm and collecting the emission spectra between 510 nm and 680 nm. Fluorescence intensity at 519 nm and 666 nm for donor and acceptor, respectively, were plotted as a function of urea concentration and the data were fitted to the following equation assuming a two-state behaviour:

$$F = \frac{(\alpha_{\mathrm{N}} + \beta_{\mathrm{N}} D) + (\alpha_{\mathrm{U}} + \beta_{\mathrm{U}} D)\exp\left(\frac{-m(\mathrm{D}_{50} - \mathrm{D})}{RT}\right)}{1 + \exp\left(\frac{-m(D_{50} - \mathrm{D})}{RT}\right)}$$

$F$ is the observed fluorescence intensity; $\alpha_N$ is the native fluorescence signal at 0 M denaturant, $\beta_N$, the slope of the baseline for the native fluorescence, $\alpha_u$ is the denatured fluorescence with the sloping baseline for the denatured protein fluorescence $\beta_u$. $D_{50}$ is the denaturant concentration at the midpoint for transition and $m$ is the $m$-value.

For ANK3-5 and ANK9-12, we observed an increase in the fluorescence at 519 nm and a decrease in the fluorescence at 666 nm upon addition of urea, as expected for a decrease in FRET between the two fluorophores upon unfolding. The $m$-values and midpoints, obtained from fitting the data to the two-state equation, are listed in Table S2. The midpoints of unfolding are similar to those listed in Table S1 measured by intrinsic protein fluorescence for the unfolding of the relevant subdomain (N-subdomain in the case of ANK3-5 and C-subdomain in the case of

ANK9-12) in the unlabeled proteins, indicating that the dyes do not greatly perturb the stability of the protein. For ANK5-8 a single transition was observed at both wavelengths but the midpoints were slightly different for the two (Fig. S1D); the midpoint obtained at 519 nm is ~0.6 M higher than those of the two unfolding transitions of unlabelled ANK5-8, suggesting that this variant is slightly stabilised by the fluorophores. For ANK8-12 the fluorescence at 666 nm decreased upon unfolding, as observed for ANK3-5 and ANK9-12; however the fluorescence at 519 nm decreased slightly at ~2 M urea and then increased at higher urea concentrations. This behavior may reflect some local rearrangement of structure around the fluorophores before the full unfolding of the protein.

*Time-resolved fluorescence measurements of labeled D34 variants, and relative orientation of the FRET-pair dyes*

Fluorescence decay traces of single- and double-labeled D34 mutants in solution, at increasing concentrations of urea, were recorded in single photon timing (SPT) mode using a FluoTime 200 fluorescence lifetime spectrometer (PicoQuant GmbH, Berlin, Germany). The excitation source was either a 485-nm pulsed laser (LDH-P-C-485, PicoQuant) for the AF488 dye, or a 632-nm pulsed laser (LDH-P-C-635B, PicoQuant) for the AF647 dye. Both lasers were controlled by a PDL-800 driver unit (PicoQuant) using a 20 MHz repetition rate. The spectral bandwidth of the lasers was ± 5 nm and the temporal full width at half maxima of the pulses was lower than 90 ps. Fluorescence was collected after crossing through a polarizer set at the magic angle and a 2-nm bandwidth monocromator. The photon events are time stamped using a TimeHarp 200 PC-board (PicoQuant) and fluorescence decay histograms plotted over 1320 channels with a time increment per channel of 36 ps. Three fluorescence decay traces with 20,000 counts at the peak channel were collected for each dye at the following emission wavelengths: 515 nm, 525 nm, and 535 nm for the AF488, and 665, 670, and 675 nm for the AF647. Decay traces were analyzed by a least squares based deconvolution method in terms of multi-exponential functions, employing instrument response functions collected using Ludox scatterer. The three different traces were fitted globally with the decay times linked as shared parameters. The quality of the fits was judged by the value of the reduced $\chi^2$ and the randomness in the distributions of weighted residuals and autocorrelation functions.

For the lifetime measurements we expressed and purified AF488 single-labeled variants with only one cysteine residue in each construct (referred to as C475,

C530, C655, C665, C764 and C780). Double-cysteine variants labeled with AF488 and AF647 were also analysed. The data are shown in figures S4 and S5.

Lifetime measurements of the AF488-labelled single-cysteine variants were performed as a function of urea concentration. This allows us to determine whether there are significant quenching effects from the neighbouring residues that could affect the FRET efficiency and thereby complicate the analysis. The lifetime for C764 (ANK 12) is unaltered upon unfolding (Fig. S2A). For C655 (ANK 8), C530 (ANK 5) and C780 (ANK12) the dye is slightly protected when folded and upon unfolding the lifetime decreases slightly, plateauing at ~3 M urea (Fig. S2A). The slight quenching of the dyes in these positions should not affect the FRET measurements. The largest effect of quenching is observed in C475 (a natural cysteine residue in D34, located in ANK 3) under native conditions. Although the quenching effect in this position is more noticeable than the others, the expected FRET towards the acceptor at cysteine C530 (the other natural cysteine residue) is much more efficient than the quenching. The competing nature of the two deactivation processes means that the faster process dominates, in this case the energy transfer. Therefore, the effect of quenching on the FRET efficiency is not very important.

The fluorescence lifetime of the AF647 dye in the folded and unfolded states was tested in the double-labelled FRET constructs (Fig. S2B). For ANK5-8 the lifetime is not altered upon unfolding. For ANK9-12 the acceptor dye, AF647, seems to be more protected in the folded state, and the lifetime is around 1.6 ns. Upon unfolding the lifetime decreases to 1.51 ns. In ANK3-5 and ANK8-12 the acceptor dye is slightly quenched in the folded state. The plots of the lifetimes together show that the environment of the dye is slightly different in the native states of different mutants, but once unfolded, AF647 has the same lifetime in all the mutants. In any case, the effects over the AF647 fluorescence lifetime are small, and the dye's ability to act as energy acceptor is not compromised by additional quenching effects.

The fluorescence lifetime of the AF488 energy donor in the double-labelled constructs was also measured as a function of urea concentration (Fig. S2C). In all constructs the donor dye AF488 is quenched by FRET when the protein is folded. The lifetime increases upon unfolding, as expected for the decreased FRET efficiency. The constructs ANK5-8 and ANK8-12 show lower quenching, consistent with the lower FRET efficiency and larger distance between the dyes. The lifetime of AF488 in the native states of ANK5-8 and ANK8-12 is 2.24 ns and 2.59 ns, respectively. Upon unfolding the lifetimes increase to 3.10 ns and 3.27 ns, respectively. These

values are lower than those of single-labeled mutants in 7 M urea (between 3.5 ns and 3.8 ns), suggesting that there is still some detectable FRET efficiency in the unfolded state. ANK9-12 and ANK3-5 show much more quenching, consistent with the high FRET in these pairs. The lifetime of AF488 in the native states of ANK9-12 and ANK3-5 is 1.01 ns and 0.97 ns, respectively. The lifetime increases upon unfolding to 3.15 ns and 2.70 ns, respectively. These values also show some detectable FRET efficiency in the unfolded state.

Due to the decrease of the donor fluorescence lifetime caused by energy transfer, the apparent FRET efficiency can be calculated from the average lifetimes. However, the double-labeled constructs have two different labeling positions for the donor and acceptor dyes, and a non-specific labeling strategy was used, so the donor fluorophore may be in different environments in different molecules. Assuming we have 50% of AF488 in one position and 50% in the other position, and given that we have measured the AF488 lifetimes in each position in the single-cysteine variants, then the average lifetime of AF488 in the absence of FRET can be determined from the average of the lifetimes obtained for the two AF488-labeled single-cysteine variants. This average lifetime in the absence of FRET ($\tau^{SL}$) can then compared with the actual average lifetime of AF488 in the double-labeled construct ($\tau^{DL}$) and the FRET efficiency calculated as: $E=1-(\tau^{DL})/(\tau^{SL})$. These results are shown in Figure S5.

Fluorescence anisotropy measurements were performed on a JASCO FP-6500 spectrofluorometer using a 450-W xenon lamp as the excitation source. Linearly polarized light of 488 and 633 nm was used for the excitation of the Alexa Fluor 488 and Alexa Fluor 647 respectively. The fluorescence anisotropy, r, is given by:

$$r = \frac{I_{VV} - G \times I_{VH}}{I_{VV} + 2G \times I_{VH}}$$

and the correction factor:

$$G = \frac{I_{HV}}{I_{HH}}$$

where the subscripts *V* and *H* represent respectively the vertical or horizontal position of the excitation (first subscript) or emission polarizer (second subscript). The obtained values of the fluorescence anisotropy are listed in Table S3.

The anisotropy values are employed to check whether the consideration of the $\kappa^2$ value of 2/3 (for random orientation of the dyes' dipoles in the FRET process) is correct into the estimation of the $R_0$ value. This is important to directly relate the FRET efficiency values with actual distances.

The Perrin equation relates the steady-state anisotropy of a fluorophore to the rotational correlation time and the lifetime of the dye. We have used this equation to estimate the limit anisotropy values for the fluorophores when attached rigidly to the protein. Considering a globular protein of 46.5 kDa, the rotational correlation time would be 18.1 ns. Applying the Perrin equation, the limit anisotropy (rigid dyes) would be 0.33, 0.35, and 0.37, respectively for fluorophores with a lifetime of 4 (AF488), 2.6 (AF488 in ANK3), and 1.5 ns (AF647). Hence, the anisotropy values of the AF488 constructs are in agreement with a depolarization occurring 5-10 fold faster than the rigid dye state. Similarly, the anisotropy values of the AF647 constructs are in agreement with a depolarization occurring 4-8 fold faster than the rigid dye state. However, the D34 is far from being a globular protein, and its shape is consistent with an even slower rotational diffusion. For a prolate ellipsoid-shaped hydrated protein with semi-axes of 65 Å and 20 Å, a much closer model for our D34 construct than the globular shape, the average of the three different rotational correlation times is 52.2 ns. With this rotational correlation time, the Perrin equation yields limit anisotropy values of 0.37, 0.38 and 0.39 for the three lifetimes mentioned above, respectively. In such case, the experimental anisotropy values indicate that the AF488 dye undergoes rotation 14-30 fold faster than the overall protein, and that the AF647 dye shows rotational diffusion 10-20 fold faster than the protein. These results are convincing enough to assume that the orientation of the dipoles in the FRET constructs can be considered random, and the $\kappa^2$ value of 2/3 is justified.

**Table S1, related to Experimental Procedures.** *m*-values and midpoints of unfolding (D50) for unlabeled wild-type D34 and variants, determined by globally fitting the denaturation curves obtained from plotting tryptophan fluorescence at 0.5 nm intervals between 320 nm and 370 nm emission wavelength to a three-state equation.

| FRET variant | Mutations | $m_{N \to I}$ (kcal mol$^{-1}$ M$^{-1}$) | D50$_{N \to I}$ (M) | $m_{I \to U}$ (kcal mol$^{-1}$ M$^{-1}$) | D50$_{I \to U}$ (M) |
|---|---|---|---|---|---|
| ANK3-5 | Wild type | 3.46±0.75 | 2.64±0.06 | 1.69±0.26 | 2.32±0.11 |
| ANK5-8 | C475S, S655C | 2.92±0.18 | 2.23±0.04 | 2.11±0.07 | 2.30±0.04 |
| ANK8-12 | C475S, C530S, S655C, S780C | 5.80±0.35 | 2.40±0.02 | 0.71±0.08 | 2.00±0.04 |
| ANK9-12 | C475S, C530S, S665C, S764C | 2.25±0.06 | 2.37±0.03 | 1.76±0.03 | 2.30±0.04 |
| ANK9-12Δ* | C475S, C530S, S665C, S764C, S799stop | 1.39±0.03 | 1.32±0.03 | | |

*For ANK9-12Δ, only one transition could be clearly detected and therefore the data were fitted to a two-state equation.

**Table S2, related to Figure 1.** *m*-values and midpoints of unfolding (D50) for double-labeled wild-type D34 and variants, determined by fitting the denaturation curves obtained from plotting fluorescence intensity at emission wavelengths of 519 nm and 666 nm (for donor and acceptor dyes, respectively) upon excitation at 496 nm to a two-state equation.

| Variant | *m* (519 nm) (kcalmol$^{-1}$M$^{-1}$) | D50 (519 nm) (M) | *m* (666 nm) (kcalmol$^{-1}$M$^{-1}$) | D50 (666 nm) (M) |
|---|---|---|---|---|
| ANK3-5 | 3.32±0.32 | 2.22±0.02 | 3.65±0.45 | 2.23±0.23 |
| ANK5-8 | 2.88±0.78 | 2.88±0.07 | 2.27±0.26 | 2.28±0.04 |
| ANK8-12* | - | - | 2.10±0.18 | 2.30±0.04 |
| ANK9-12 | 2.24±0.13 | 2.46±0.02 | 2.38±0.20 | 2.33±0.03 |
| ANK9-12Δ* | 4.04±2.0 | 1.95±0.23 | - | - |

*For ANK8-12, only the 666 nm data, for which there was a single transition, were fitted. For ANK9-12Δ, only the 519 nm data gave reliable fitting values, whereas the signal to noise for the 666 nm data was too low for a reliable fit.

**Table S3, related to Figure 1.** Anisotropy values of single- and double-labeled proteins.

| Labeled residue | Location | *Anisotropy (r) donor* | *Anisotropy (r) acceptor* |
|---|---|---|---|
| C475, C530 | ANK 3, 5 | 0.08 | 0.24 |
| C530, C655 | ANK 5, 8 | 0.17 | 0.25 |
| C655, C780 | ANK 8, 12 | 0.08 | 0.26 |
| C665, C764 | ANK 9, 12 | 0.13 | 0.30 |
| C475 | ANK 3 | 0.24 | - |
| C530 | ANK 5 | 0.16 | - |
| C655 | ANK 8 | 0.13 | - |
| C665 | ANK 9 | 0.13 | - |
| C764 | ANK 12 | 0.17 | - |
| C780 | ANK 12 | 0.12 | - |

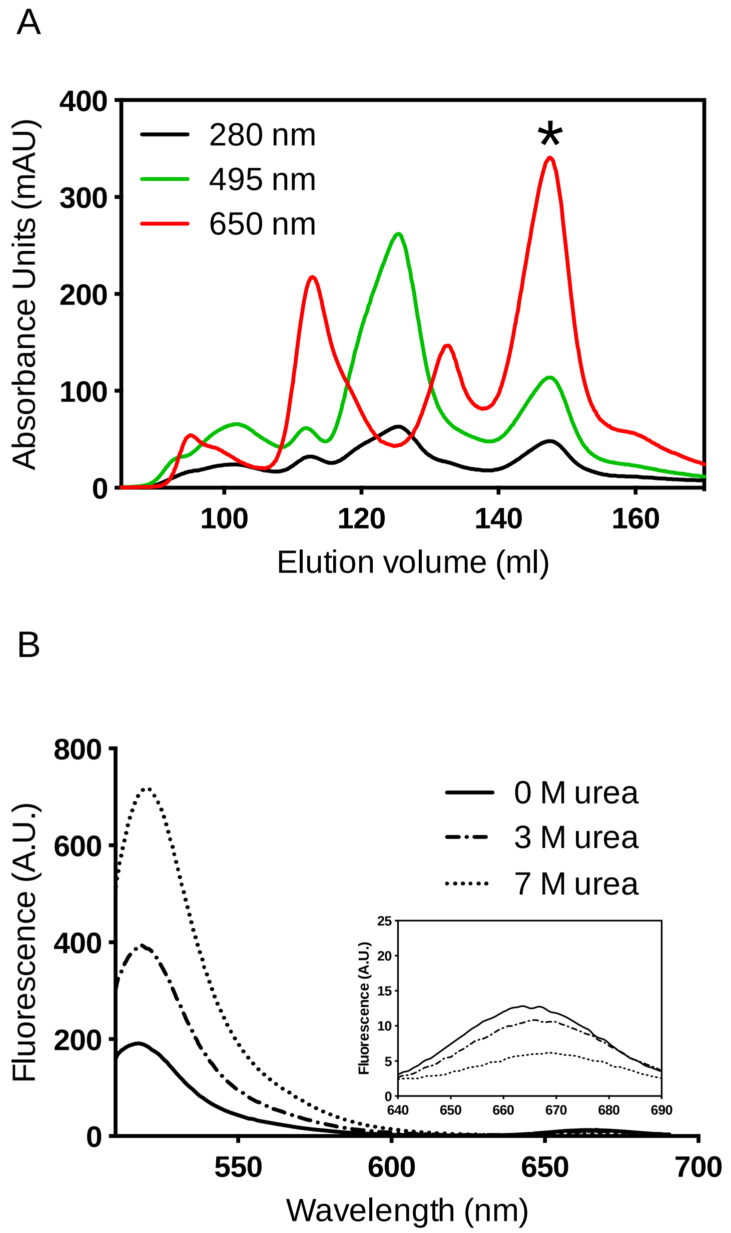


**Figure S1, related to Experimental Procedures: Purification of AF488- and AF647-double-labelled wild-type D34.**

(A) Wild-type D34 was labelled sequentially with AF647 and AF488. Unlabelled, homo-labelled (AF647 only or AF488 only) and hetero-labelled (AF488 and AF647 present) were separated by anion-exchange chromatography (MonoQ, GE Healthcare). The absorbance was followed at three wavelengths simultaneously (280 nm, 495 nm and 650 nm). The fractions containing D34 with both dyes were collected and pooled (indicated by a star).

(B) Fluorescence emission spectra of double-labeled wild-type D34 in 0 M, 3 M and 7 M urea using an excitation wavelength of 495 nm (inset shows the region around 666 nm magnified). Measurements were made at a protein concentration of 100 nM concentration in 50 mM Tris-HCl buffer pH 8, 150 mM NaCl and 1 mM TCEP at 25 °C.

C

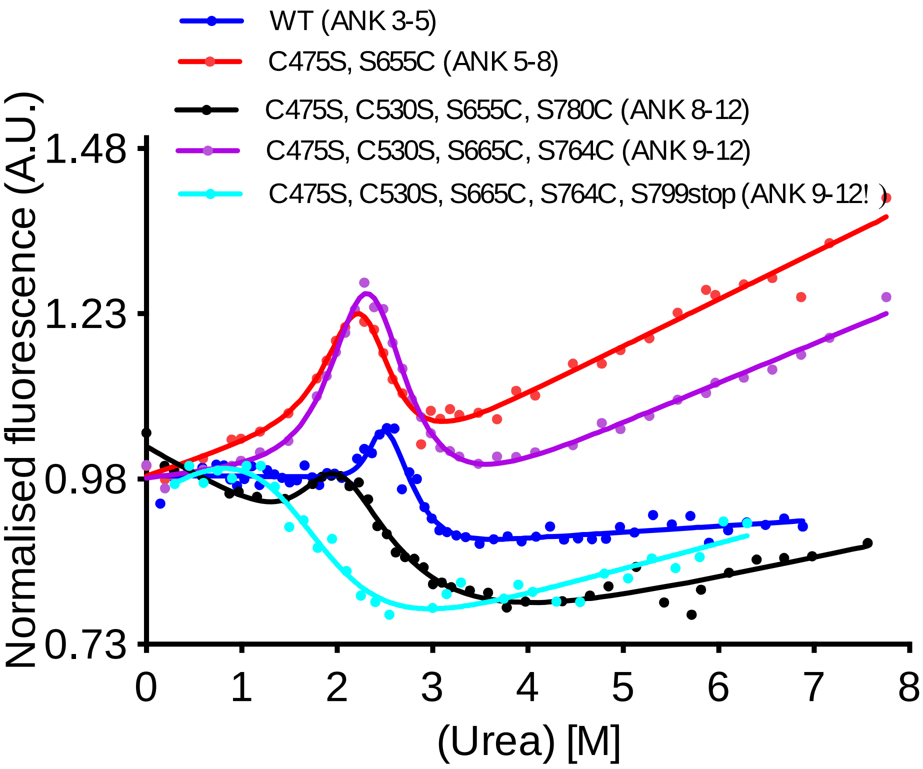


(C) Ensemble measurements of urea-induced denaturation for (unlabelled) D34 wild type and cysteine mutants monitored by tryptophan fluorescence. Denaturation profiles of WT (blue) and double-cysteine mutants of D34, monitored by fluorescence at 365 nm using an excitation wavelength of 280 nm. The data were collected at 1 µM protein concentration in 50 mM Tris pH-HCl buffer pH 8, 150 mM NaCl and 1 mM DTT at 25 °C. Wild-type D34 (WT) has cysteines at position 475 and 530 and is referred to here as ANK3-5 (blue); ANK5-8 has mutations C475S, S655C (red); ANK9-12 has C475S, C530S, S665C, S764C (purple); ANK8-12 has C475S, C530S, S655C, S780C (black). The denaturation curves were fitted to a three-state equation as described in the SI.

D

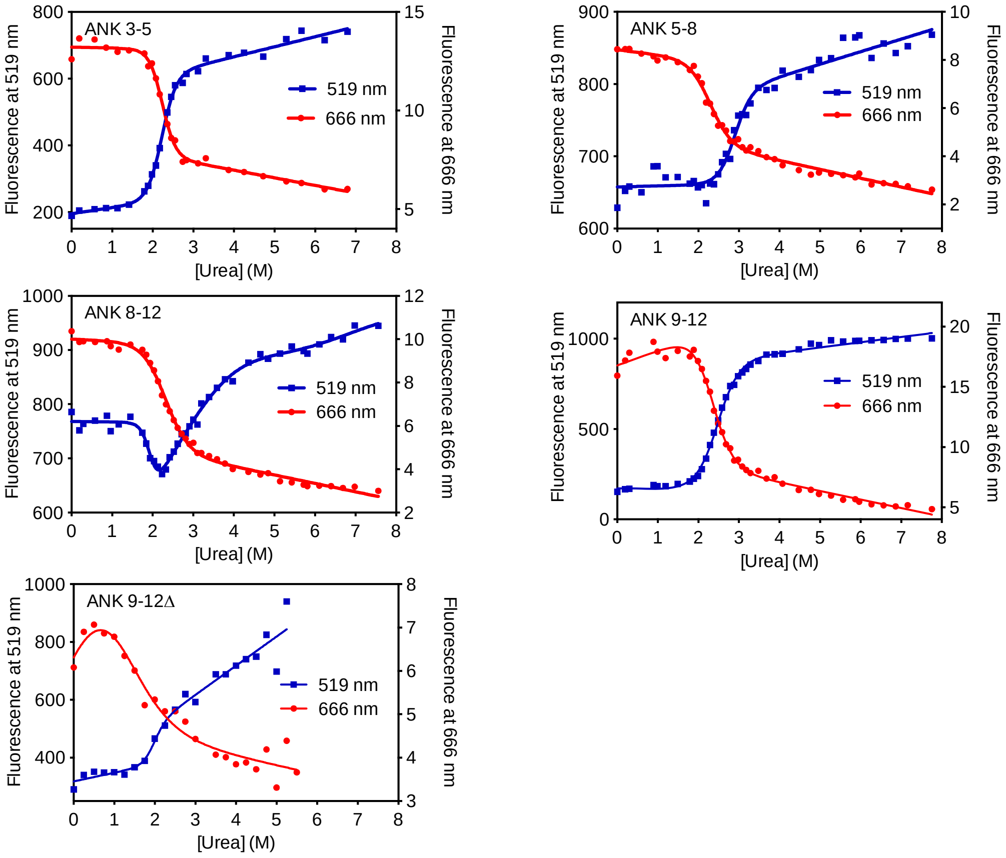


(D) Urea-induced denaturation profiles of D34 variants measured by AF488 and AF647 fluorescence. The data were collected at 0.2 μM protein concentration in 50 mM Tris-HCl buffer pH 8, 150 mM NaCl and 1 mM DTT at 25 °C. The denaturation curves were fitted to a two-state equation, with the exception of that of ANK8-12 at 519 nm, which was fitted to a three-state equation. The data for ANK9-12Δ at 666 nm gave high fitting errors due to the low signal-to-noise.

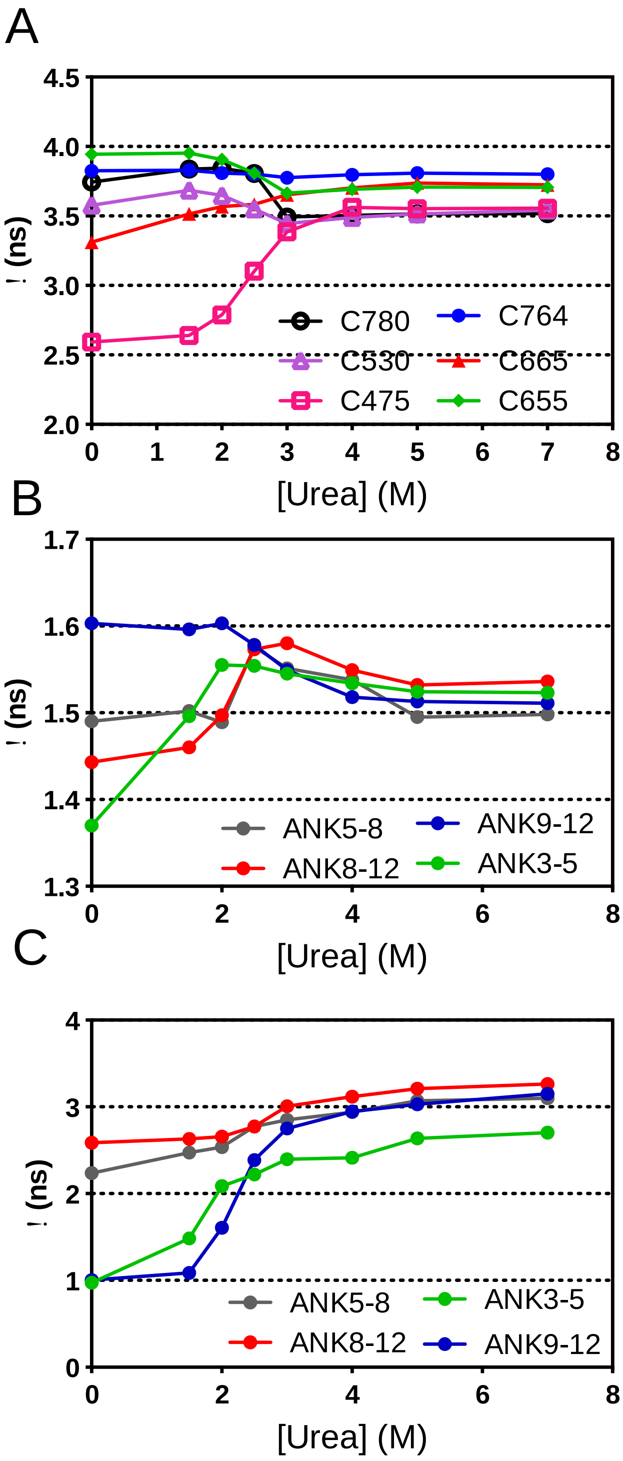


**Figure S2, related to Figure 1. AF488 and AF647 fluorescence lifetimes in the single-labeled single-cysteine and double-labeled double-cysteine constructs as a function of increasing urea concentration**

(A) AF488 fluorescence lifetimes in the single-cysteine constructs.

(B) AF647 and (C) AF488 fluorescence lifetimes in the double-cysteine constructs.

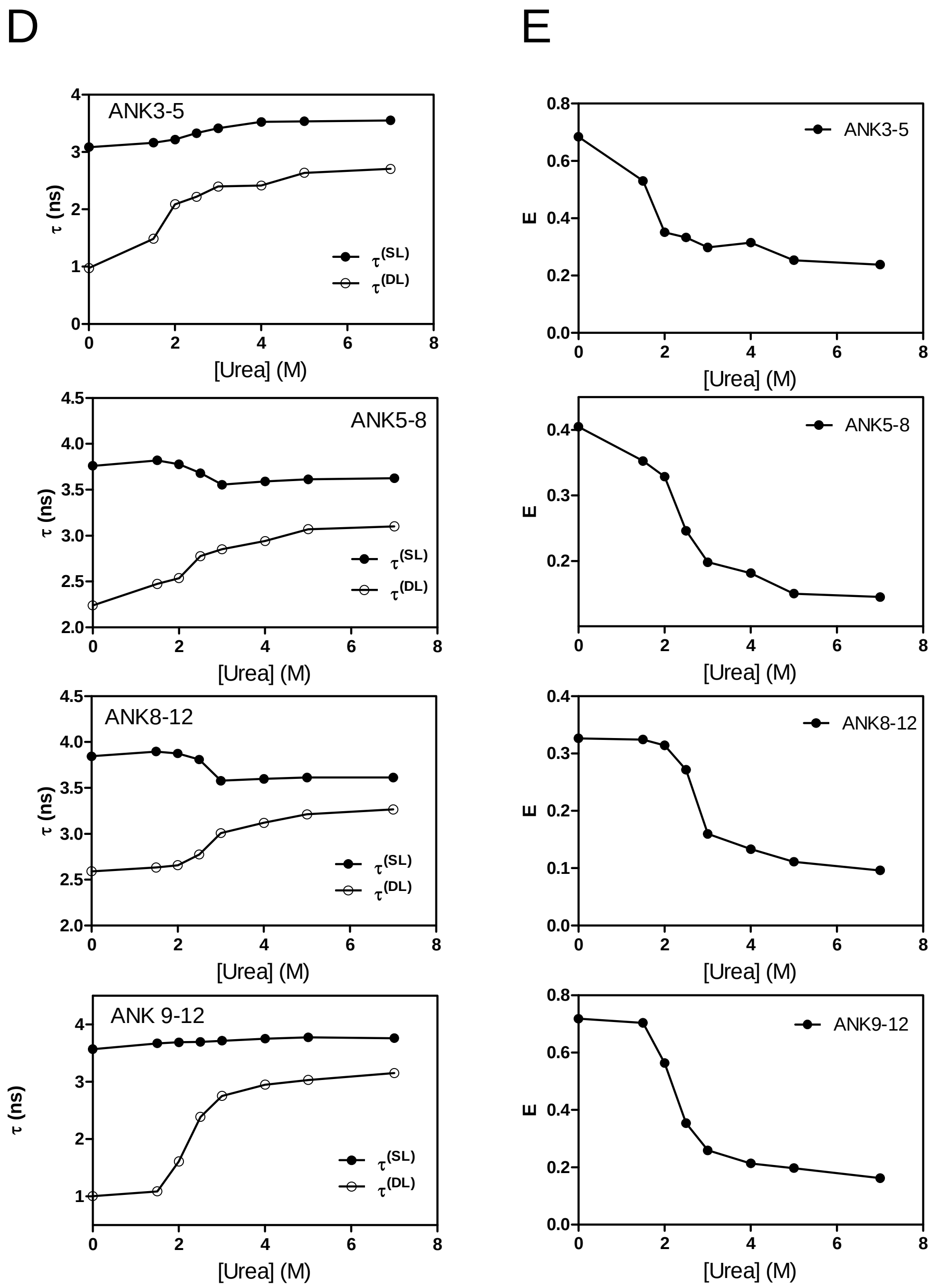


AF488 fluorescence lifetimes and FRET efficiencies of double-cysteine constructs as a function of urea concentration. (D) The average AF488 fluorescence lifetime $\tau^{SL}$ is the mean of the AF488 fluorescence lifetimes in the two single-labeled single-cysteine constructs. $\tau^{DL}$ is the AF488 fluorescence lifetime in the double-labeled double-cysteine constructs.

(E) The FRET efficiency, E, is calculated from $\tau^{SL}$ and $\tau^{DL}$ using the following equation: $E=1-(\tau^{DL})/(\tau^{SL})$.

A

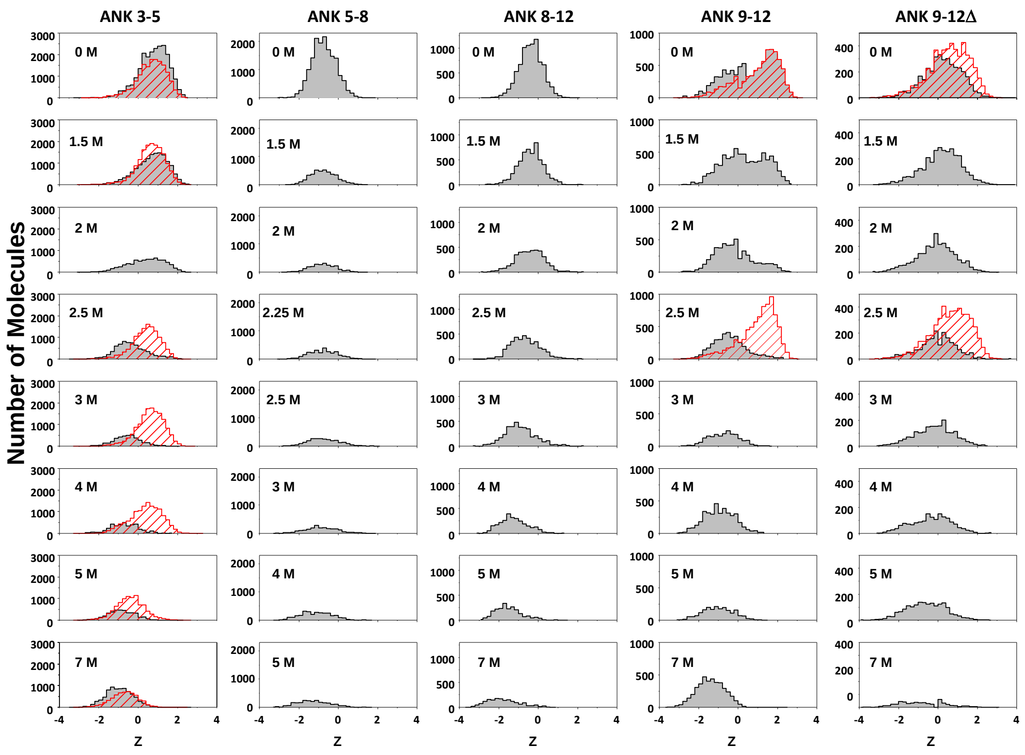


B

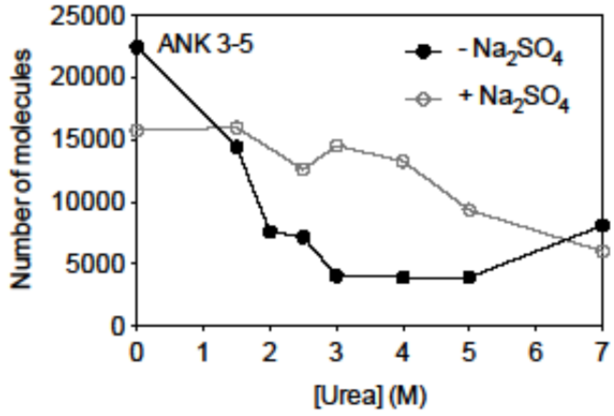


**Figure S3, related to Figure 2.** (A) Urea titrations of the FRET Z-parameter histograms of labelled wild-type D34 (ANK3-5) and variants. The data obtained for ANK3-5, ANK9-12 and ANK9-12Δ in the presence of 400 mM $Na_2SO_4$ are shown in red. (B) Comparison of the number of molecules as measured by 1 color FRET excitation *versus* urea concentration for ANK3-5 in the presence and absence of 400 mM $Na_2SO_4$.

## References

Clarke, R.W., Orte, A., and Klenerman, D. (2007). Optimized threshold selection for single-molecule two-color fluorescence coincidence spectroscopy. Analytical chemistry *79*, 2771-2777.

Werbeck, N.D. and Itzhaki, L.S. (2007). Probing a moving target with a plastic unfolding intermediate of an ankyrin-repeat protein. Proceedings of the National Academy of Sciences of the United States of America *104*(19), 7863-7868.
